# Supplementary material for: Multi-Level Biomarkers for Early Diagnosis of Ischaemic Stroke: A Systematic Review and Meta-Analysis
Source: Int J Mol Sci. 2023 Sep 7;24(18):13821. doi: 10.3390/ijms241813821 (PMC10530879; doi:10.3390/ijms241813821)
Supplement: Supplementary file 1 [file ijms-24-13821-s001.zip › ijms-2574458-supplementary.pdf]

Multi-level blood biomarkers for early diagnosis of ischaemic stroke: a systematic review and meta-analysis

Qianyun Li[1], Lingyun Zhao[1], Ching Long Chan[1], Yilin Zhang[1], See Wai Tong[1], Xiaodan Zhang[1], Joshua Wing Kei Ho[2], Yaqing Jiao[1], Timothy Hudson Rainer*[1]

[1]Department of Emergency Medicine, University of Hong Kong, Hong Kong SAR, China

[2]School of Biomedical Sciences, University of Hong Kong, Hong Kong SAR, China

Index of appendices

Supplementary Table 1: Search strategies ……………………………………………………………………………….2

Supplementary table 2: Concise characteristics of 182 included studies………………………………………………………6

Supplemental Table 3: Brain-specific or enriched biomarkers for diagnosis of ischaemic stroke in early stage……………………..…17

Supplementary Table 4: Characteristics of included high-throughput omics studies…………………………………………………20

Supplementary List 5: List of omics biomarkers pooled from high-throughput omics studies ………………………………………24

Supplementary Table 6: Quality assessment results using QUADAS-2……………………………………………………………...26

Supplementary Table 7: Characteristics of included 10 studies for diagnostic meta-analysis………………………………………..33

References……………………………………………………………………………………………........................34

Supplementary Table 1: Search strategies

PubMed: 1971 to 4 June 2022

#1	Blood[MeSH] OR blood[Title/Abstract] OR plasma[Title/Abstract] OR serum[Title/Abstract]
#2	“Biomarkers”[MeSH] OR biomarker*[Title/Abstract] OR “biological marker*”[Title/Abstract] OR “biologic marker*”[Title/Abstract] OR “serum marker*”[Title/Abstract] OR “biochemical marker*”[Title/Abstract] OR “laboratory marker*”[Title/Abstract]
#3	Diagnosis[MeSH] OR diagnos*[Title/Abstract] OR differentiat*[Title/Abstract]
#4	Stroke[MeSH] OR “stroke*”[Title/Abstract] OR “cerebrovascular accident*”[Title/Abstract] OR “brain vascular accident*”[Title/Abstract] OR “brain infarct*”[Title/Abstract] OR “intracerebral hemorrhage*”[Title/Abstract] OR “cerebral hemorrhage*”[Title/Abstract]
#5	1 AND #2 AND #3 AND #4

Ovid Embase: 1974 to 4 June 2022

#1	'blood'/exp
#2	blood:ab,ti OR plasma:ab,ti OR serum:ab,ti
#3	#1 OR #2
#4	'biological marker'/exp
#5	biomarker:ab,ti OR biomarkers:ab,ti
#6	biologic:ab,ti AND marker:ab,ti
#7	biologic:ab,ti AND markers:ab,ti
#8	biochemical:ab,ti AND marker:ab,ti
#9	biochemical:ab,ti AND markers:ab,ti
#10	laboratory:ab,ti AND marker:ab,ti
#11	laboratory:ab,ti AND markers:ab,ti
#12	#4 OR #5 OR #6 OR #7 OR #8 OR #9 OR #10 OR #11

#13	'diagnosis'/exp
#14	diagnosis:ab,ti OR diagnoses:ab,ti OR diagnose:ab,ti OR diagnostic:ab,ti OR diagnostics:ab,ti OR diagnostically:ab,ti
#15	differentiation:ab,ti OR differentiate:ab,ti OR differentiating:ab,ti OR differentiability:ab,ti
#16	#13 OR #14 OR #15
#17	'cerebrovascular accident'/exp
#18	stroke:ab,ti OR strokes:ab,ti
#19	cerebrovascular:ab,ti AND accident:ab,ti
#20	cerebrovascular:ab,ti AND accidents:ab,ti
#21	brain:ab,ti AND vascular:ab,ti AND accident:ab,ti
#22	brain:ab,ti AND vascular:ab,ti AND accidents:ab,ti
#23	brain:ab,ti AND infartion:ab,ti
#24	brain:ab,ti AND infart:ab,ti
#25	cerebral:ab,ti AND hemorrhage:ab,ti
#26	intracerebral:ab,ti AND hemorrhage:ab,ti
#27	#17 OR #18 OR #19 OR #20 OR #21 OR #22 OR #23 OR #24 OR #25 OR #26
#28	#3 AND #12 AND #16 AND #27

Cochrane library: 1993 to 4 June 2022

#1	MeSH descriptor: [Blood] this term only
#2	MeSH descriptor: [Plasma] this term only
#3	MeSH descriptor: [Serum] this term only
#4	(blood):ti,ab,kw OR (plasma):ti,ab,kw OR (serum):ti,ab,kw in Trials (Word variations have been searched)
#5	#1 OR #2 OR #3 OR #4
#6	MeSH descriptor: [Biomarkers] this term only
#7	(biomarker):ti,ab,kw OR (biomarkers):ti,ab,kw in Trials (Word variations have been searched)
#8	(biological):ti,ab,kw OR (biologic):ti,ab,kw OR (biomedical):ti,ab,kw OR (laboratory):ti,ab,kw in Trials (Word variations have been searched)

#9	(marker):ti,ab,kw OR (markers):ti,ab,kw in Trials (Word variations have been searched)
#10	#8 AND #9
#11	#6 OR #7 OR #10
#12	MeSH descriptor: [Diagnosis] this term only
#13	(diagnoses):ti,ab,kw OR (diagnose):ti,ab,kw OR (diagnostic):ti,ab,kw OR (diagnostics):ti,ab,kw OR (diagnostically):ti,ab,kw in Trials (Word variations have been searched)
#14	(differentiate):ti,ab,kw OR (differentiation):ti,ab,kw OR (differentiating):ti,ab,kw OR (differentiability):ti,ab,kw in Trials (Word variations have been searched)
#15	#12 OR #13 OR #14
#16	MeSH descriptor: [Stroke] this term only
#17	(stroke):ti,ab,kw in Trials (Word variations have been searched)
#18	(cerebrovascular):ti,ab,kw AND (accident):ti,ab,kw in Trials (Word variations have been searched)
#19	(brain):ti,ab,kw AND (vascular):ti,ab,kw AND (accident):ti,ab,kw in Trials (Word variations have been searched)
#20	(brain):ti,ab,kw AND (infarction):ti,ab,kw in Trials (Word variations have been searched)
#21	(cerebral):ti,ab,kw AND (hemorrhage):ti,ab,kw in Trials (Word variations have been searched)
#22	(intracerebral):ti,ab,kw AND (hemorrhage):ti,ab,kw in Trials (Word variations have been searched)
#23	#16 OR #17 OR #18 OR #19 OR #20 OR #21 OR #22 in Trials
#24	#5 AND #11 AND #15 AND #23 in Trials

Web of science: 1900 to 4 June 2022

#1	blood (Topic) or plasma (Topic) or serum (Topic)
#2	biomarkers (Topic) or biomarker (Topic)
#3	biological (Topic) and marker (Topic)
#4	biologic (Topic) and marker (Topic)

#5	biochemical (Topic) and marker (Topic)
#6	laboratory (Topic) and marker (Topic)
#7	#2 OR #3 OR #4 OR #5 OR #6
#8	diagnosis (Topic) or diagnoses (Topic) or diagnose (Topic) or diagnostic (Topic) or diagnostics (Topic) or diagnostically (Topic)
#9	differentiate (Topic) or differentiation (Topic) or differentiating (Topic) or differentiability (Topic)
#10	#8 OR #9
#11	Stroke (Topic)
#12	cerebrovascular (Topic) and accident (Topic)
#13	brain (Topic) and vascular (Topic) and accident (Topic)
#14	brain (Topic) and infarction (Topic)
#15	brain (Topic) and infarct (Topic)
#16	cerebral (Topic) and hemorrhage (Topic)
#17	intracerebral (Topic) and hemorrhage (Topic)
#18	#11 OR #12 OR #13 OR #14 OR #15 OR #16 OR #17
#19	#1 AND #7 AND #10 AND #18

Supplementary Table 2: Concise characteristics of 182 included studies

Study ID	Country	Design	Comparison	Setting	Reference	Specimen	Sampling time	Omics	Biomarkers
Zhou 2021 (1)	China	Case control study	IS vs. Controls	Not stated	CT; MRI	serum	24h	transcripts	miR-124
Zhang 2022 (2)	China	Case control study	IS vs. Controls	Not stated	Not stated	serum	9h	proteins	JKAP
Yang 2022 (3)	China	Case control study	IS vs. Controls	Not stated	Not stated	plasma; exosome	12h	Transcriptomics	circ_0112036, circ_0066867, circ_0093708, circ_0041685
Turek-Jakubowska 2022 (4)	Poland	Case control study	IS vs. Controls	Neurology ward	CT	plasma	24h	Proteomics	Alpha-1B-glycoprotein
Tian 2022 (5)	China	Case control study	IS vs. Controls	Neuro-vascular centre	Not stated	whole blood	6h	Transcriptomics	lncRNA NR_120420
Rahmati 2021 (6)	Iran	Case control study	IS vs. Controls	Not stated	MRI	serum	24h	transcripts; proteins	miR-210, HIF-1a
Li 2021 (7)	China	Case control study	IS vs. Controls	Not stated	CT; MRI	serum	24h	proteins; metabolites	uric acid, CRP, NT-proBNP
Induruwa 2022 (8)	UK	Prospective study	IS vs. Controls; HS vs. Controls; Total stroke vs. controls	ED	Not stated	whole blood; platelet	8h	proteins; platelet	IS vs. control: GPVI-dimer, HS vs. control: GPVI-dimer, total stroke vs. control: GPVI, GPVI-dimer, platelet P-selectin
Gawryś 2022 (9)	Poland	Case control study	IS vs. Controls	Neurology ward	CT	platelet	24h	Proteomics; platelet	Beta-amyloid protein A4, Amyloid-like protein 2, coactosin-like protein, thymidine phosphorylase 4 (TYMP-4), interferon regulatory factor 7 (IRF7), vitamin K-dependent protein S, histone proteins (H2A type 1 and 1-A, H2A types 2B and J, H2Av, -z, and -x), platelet basic protein
Cho 2022 (10)	not stated	Case control study	IS vs. Controls	ED	Not stated	PBMCs	24h	PBMC number	NK cells, CD14+ monocytes
Intiso 2004 (11)	Italy	Prospective study	IS vs. Controls	Neurology ward	CT; MRI	serum	24h	proteins	TNFa
Perini 2001 (12)	Italy	Prospective study	IS vs. Controls	Stroke Unit	CT	serum	12h	proteins	IL-6, IL-10
Pedersen 2004 (13)	Norway	Case control study	IS vs. Controls	Not stated	CT; MRI	plasma	24h	proteins	CRP
Nayak 2011 (14)	India	Case control study	IS vs. Controls	Not stated	CT	serum	24h	proteins	IMA
Senes 2007 (15)	Turkey	Prospective study	IS vs. Controls	Not stated	Not stated	serum	24h	metabolites	nitrite, nitrate, IMA, TBARS
Feng 2019 (16)	China	Case control study	IS vs. Controls	Not stated	CT; MRI	plasma	24h	transcripts	lncRNA ANRIL
Blann 1999 (17)	UK	Prospective study	IS vs. Controls	Not stated	Not stated	serum; plasma	12h	proteins	ICAM-1, E-selectin, VCAM-1, vWF
Shyu 1997 (18)	China (Taiwan)	Prospective study	IS vs. Controls	ED	CT	serum	24h	proteins	ICAM-1, E-selectin

Liu 2015 (19)	China	Prospective study	IS vs. Controls	Not stated	CT; MRI	serum	24h	proteins	CXCL12
Supanc 2011 (20)	Croatia	Prospective study	IS vs. Controls	Neurology ward	CT	serum	24h	proteins	ICAM-1, VCAM-1
Wunderlich 2005 (21)	Germany	Prospective study	IS vs. Controls	Neurology ward	CT	serum	24h; 18h; 12h; 6h; 3h; 2h; 1h	proteins	B-FABP, H-FABP
Hu 2016 (22)	China	Case control study	IS vs. HS	Not stated	Not stated	whole blood	12h	metabolites	Asn, C5:1, Arg/Orn, Val/Phe, (C0 +C2 + C3 + C16 + C18:1)/Cit
Uno 2003 (23)	Japan	Prospective study	IS vs. Controls	department of neurological surgery	CT; MRI	plasma	24h	proteins	OxLDL
Sun 2019 (24)	Germany	Prospective study	IS vs. Controls	ED	CT; MRI	serum	24h	Metabolomics	tetradecanedioate, hexadecanedioate
Song 2006 (25)	Korea	Prospective study	IS vs. Controls	Not stated	CT; MRI	plasma	24h	proteins	IL-6,PAI-1,PAP
Zitnanova 2016 (26)	Slovakia	Case control study	IS vs. Controls	Neurology ward	CT	plasma	24h	proteins	lipid peroxides, superoxide dismutase activity, catalase activity, paraoxonase activity, glutathione peroxide activity
Can 2015 (27)	Turkey	Prospective study	IS vs. Controls	ED	MRI	serum	12h	proteins	MBP, IMA
Kimberly 2013 (28)	USA	Prospective study +animal experiment study	IS vs. Controls	ED	MRI	plasma	2h; 9h	Metabolomics; targeted	BCAA (leucine, isoleucine, valine), carnitine, threonine, histidine, glucose, methionine, glycine, proline, lysine, cysteamine, uridine, 5'-adenosylhomocysteine, creatinine, N-carbamoyl-beta-alanine, cis/trans hydroxyproline, asparagine
Abboud 2007 (29)	France	Prospective study	IS vs. HS; Total stroke vs. TIA	ED	CT; MRI	serum	3h	proteins	IMA
Tang 2006 (30)	USA	Case control study	IS vs. Controls	Not stated	CT	whole blood	24h; 5h; 3h	Genomics	Hox 1.11 gene, CKAP4 gene, S100A9 gene, MMP9 gene, S100P gene, F5 gene, FPR1 gene, S100A12 gene, RNASE2 gene, ARG1 gene, CA4 gene, LY96 gene, SLC16A6 gene, HIST2H2AA gene, ets-2 gene, BCL6 gene, PYGL gene, NPL gene
Rainer 2007 (31)	China	Prospective study	IS vs. HS	ED	CT; MRI	plasma	24h	proteins; cfDNA	cfDNA, S100
Tiedt 2018 (32)	Germany	Prospective study	IS vs. Controls	ED	MRI	serum	24h	proteins	NfL
Zhu 2019 (33)	China	Case control study	IS vs. Controls	Not stated	CT; MRI	PBMC	24h	transcripts	PBMC circ-DLGAP4
Zhu 2018 (34)	China	Case control study	IS vs. Controls	Not stated	MRI	leukocytes	24h	transcripts	lncRNA MIAT
Zhou 2016 (35)	China	Prospective study	IS vs. HS	ED	CT	plasma	6h	proteins	S100B
Zhou 2018 (36)	China	Case control study	IS vs. Controls	Not stated	MRI	serum; exosome	24h	transcripts	miR-134
Zhou 2022 (37)	China	Case control +animal study	IS vs. Controls	Neurology department	CT; MRI	Serum (small extracellular vesicles)	24h	Transcriptomics	miR-9-3p, miR-124-3p, miR-143-3p, miR-93-5p

Zhao 2016 (38)	China	Prospective study	IS vs. Controls	Neurology ward	Not stated	serum	24h	proteins	Apolipoprotein A1-Unique Peptide (APOA1-UP)
Zhao 2017 (39)	China	Case control study	IS vs. Controls; HS vs. Controls	ED, neurology department	CT; MRI	plasma; neutrophils, lymphocytes	6h	transcripts	miR-99a-5p
Zhao 2016 (40)	China	Prospective study	IS vs. Controls	Neurology ward	MRI	plasma	24h	transcripts	miR-335
Zhang 2017 (41)	China	Case control study	IS vs. HS; IS vs. Controls; HS vs. Controls	Not stated	CT; MRI	dried blood spot	12h	Metabolomics	IS vs. control: C22, C5, C3DC, C4, C5DC/C5-OH, C3DC/C10, C14:2, C10:2, (0+2+3+16+18:1)/Cit, Arg, Pro HS vs. control: C16-OH/C16, C16:1-OH, C10, C5/C3, C12, C18, C18:1, C4DC, Val/Phe, C16, Arg, Thr IS vs. HS: C4-OH, C5DC, C14, C16-OH, Tyr/Cit, Val/Phe, C5DC,/C5-OH, C5DC/C16, C18-OH, (0+2+3016+18:1)/Cit, C3/Met
Zhang 2020 (42)	China	Prospective study	IS vs. Controls	Not stated	CT; MRI	plasma; endothelial micro-vesicles	24h	transcripts; endothelial microvesicles	EMVs, EMVs-miR-155
Zaremba 2006 (43)	Poland	Case control study	IS vs. Controls	Not stated	CT	serum	24h	proteins	IL-12
Yuan 2020 (44)	China	Case control study + animal experiment study	IS vs. Controls	Neurology ward	CT; MRI	plasma	24h	proteins	GMFB
Yigit 2017 (45)	Turkey	Case control study	IS vs. Controls; HS vs. Controls	ED	Not stated	serum	24h	proteins	UCH-L1
Yang 2016 (46)	China	Case control study	IS vs. Controls	Not stated	CT; MRI	plasma	24h	transcripts	miR-107, miR-128b, miR-153
Xiong 2015 (47)	China	Prospective study	IS vs. HS	Not stated	CT; MRI	serum	6h	proteins	GFAP
Wu 2020 (48)	China	Case control study	IS vs. Controls	Neurology ward	CT; MRI	plasma	6h	transcripts	miR-99b
Williams 2007 (49)	USA	Prospective study	IS vs. SM	ED	MRI	plasma	24h	Endothelial microparticles (EMPs)	number of Endothelial microparticles
Wang 2017 (50)	China	Case control study	IS vs. Controls	ED	CT; MRI	serum	6h	transcripts	miR-221-3p, miR-382-5p
Wang 2014 (51)	China	Prospective study	IS vs. Controls	Not stated	MRI	plasma	24h	transcripts	miR-106b-5P, miR-4306, miR-320e, miR-320d
Wang 2018 (52)	China	Case control study	IS vs. Controls	Neurology ward	CT; MRI	plasma; exosome	6h	transcripts	miR-21-5p, miR-30a-5p
Wang 2017 (53)	China	Case control study + animal experiment study	IS vs. Controls	Not stated	MRI	plasma; lymphocytes	3h	transcripts	lncRNA H19
Walsh 2016 (54)	USA	Case control study	IS vs. HS; IS vs. Controls; HS vs. Controls	ED, neurology departm	Not stated	plasma	12h	proteins	Apo A-I, Apo C-I, Apo C-III, MMP-3, MMP-9, paraoxonase-1

				ent, NICU					
Vukasovic 2006 (55)	Croatia	Case control study	IS vs. Controls	Not stated	CT	serum	24h	proteins	MMP-2, TIMP-2
von Recum 2015 (56)	Germany	Prospective study	IS vs. TIA	ED	Not stated	serum	4.5h	proteins	copeptin
Unden 2009 (57)	Sweden	Prospective study	IS vs. HS	Not stated	CT	not stated	24h	proteins	S100B, NSE, GFAP, APC-PCI
Tunç 2018 (58)	Turkey	Prospective study	IS vs. Controls	Not stated	CT; MRI	serum	24h	proteins	SPA
Tiedt 2017 (59)	Germany	Prospective study	IS vs. TIA; IS vs. Controls	ED	CT; MRI	plasma	24h	Transcriptomics	miR-125a-5p, miR-125b-5p, miR-143-3p
Tiedt 2020 (60)	Germany	Prospective study	IS vs. SM; IS vs. Controls	ED	CT; MRI	serum	24h	Metabolomics	asymmetrical dimethylarginine (ADMA), symmetrical dimethylarginine (SDMA), pregnenolone sulphate, adenosine
Tian 2015 (61)	China	Prospective study	IS vs. Controls	Not stated	CT; MRI	serum	24h	proteins	PCT, hsCRP, HCY
Tian 2016 (62)	China	Prospective study	IS vs. Controls	Cerebrovascular Diseases Centre	Not stated	plasma	6h	Transcriptomics	miR-16
Taema 2014 (63)	Egypt	Prospective study	IS vs. HS	Not stated	CT	serum	24h	proteins	CRP
Stejskal 2011 (64)	Czech Republic	Case control study	IS vs. Controls	Not stated	CT; MRI	serum	3h	proteins	VILIP-1
Stanca 2015 (65)	Romania	Prospective study	IS vs. HS	ED	CT	serum	24h; 12h	proteins	GFAP, antibodies against NMDA receptor subunit NR2
Stamova 2010 (66)	USA	Case control study	IS vs. Controls	Not stated	Not stated	whole blood	24h; 5h; 3h	Genomics	GENES (ABCA1, PGM5, CCDC144C /// LOC100134159, LECT2, SHOX, TBX5, SPTLC3, SNIP, RBMS3, P704P, THSD4, FAT3, SNRPN, GLYATL1, GADL1, DKFZP434L187, CXADR, OVOL2, RNF141, CLEC4E, ELL2, SPIB, BXDC5, UNC5B, TIMP2, ASTN2, FLJ35934, ANKRD28, CCDC144A, TIMM8A, ALDOAP2, LDB3, PTPRD, LOC729222 ///PPFIBP1, CCRL1, HNRNPUL2, FCRL4, ELAVL2, PRTG, DLX6, FOXA2, SCD5, GABRB2, GYPA, OSBPL1A, PHTF1, CKLF, CKLF, RRAGD, CLEC4E, CKLF, FGD4, CPEB2, LOC100290882, UBXN2B, ENTPD1, BST1,LTB4R,F5,IFRD1,KIAA0319, CHMP1B, MCTP1, VNN3, AMN1, LAMP2, FCHO2, ZNF608, REM2, QKI, RBM25, FAR2, ST3GAL6, HNRNPH2, GAB1, UBR5,VAPA,THBD,LOC283027, LOC344595,RPL22, LOC100129488, RPL22, MCTP1, SH3GL3)
Stamova 2019 (67)	USA	Case control study	IS vs. HS; IS vs. Controls; HS vs. Controls	Not stated	CT; MRI	not stated	24h	Transcriptomics	HS vs. control: 489 transcripts; IS vs. control: 396 transcripts; IS vs. HS: 256 transcripts
Song 2019 (68)	USA	Case control study	IS vs. Controls	Not stated	CT; MRI	serum	24h	Proteomics	clusterin, cystatin C (CST3)
Simats 2020 (69)	Spain	Case control study + animal	IS vs. SM	ED	Not stated	plasma	6h	Transcriptomics; Proteomics	CTNND2

		experiment study							
Simats 2018 (70)	Spain	Case control study+ animal experiment study	IS vs. Controls	Not stated	Not stated	plasma	6h	Proteomics; proteins	CKB, CMPK
Simats 2018 (71)	Spain	Prospective study + animal experiment study	IS vs. Controls	ED	Not stated	serum	6h; 4.5h	proteins	CCL23, CCL9
Sharma 2014 (72)	USA	Prospective study	IS vs. HS; IS vs. SM; Total stroke vs. SM	ED	CT; MRI	plasma	24h	proteins	eotaxin, epidermal growth factor receptor, S100A12, metalloproteinase inhibitor-4 (TIMP-4), prolactin
Sharma 2015 (73)	India	Case control study	IS vs. Controls	Not stated	CT; MRI	serum	24h	Proteomics	vWF, ADAMTS13, S100A7
Shaker 2020 (74)	Iraq	Case control study	IS vs. Controls	Not stated	CT	plasma	24h	proteins	GPBB
Sepramaniam 2014 (75)	Singapore	Prospective study+ animal experiment study	IS vs. Controls	Not stated	CT; MRI	whole blood	24h	Transcripto mics	miR-125b-2*, miR-27a*, miR-422a, miR-488, miR-627
Sayan 2016 (76)	Turkey	Prospective study	IS vs. Controls	Neurolo gy ward	CT	plasma	24h	proteins	BNP
Rozanski 2017 (77)	Germany	Prospective study	IS vs. HS	Stroke Emerge ncy Mobile	CT	plasma	3h; 1h	proteins	GFAP
Roudbary 2011 (78)	Iran	Cross sectional study	IS vs. HS	Neurolo gy ward	CT	serum	24h	proteins	hsCRP
Rico Santana 2014 (79)	Spain	Case control study	IS vs. Controls	ED, neurolo gy departm ent	CT; MRI	serum	6h	Proteomics	2155-Da peptide
Richard 2016 (80)	France	Case control study	IS vs. Controls	Not stated	CT; MRI	plasma	24h; 6h; 3h	proteins	PRDX1
Reynolds 2003 (81)	USA	Case control study	IS vs. HS; IS vs. TIA; IS vs. Controls; HS vs. TIA; HS vs. Controls; Total stroke vs. controls	ED	CT; MRI	serum	24h; 12h; 6h; 3h	proteins	S100B, BNGF, vWF, MMP9, MCP-1
Ren 2016 (82)	China	Case control study	IS vs. HS; IS vs. Controls; HS vs. Controls	ED	CT; MRI	serum	24h; 4.5h	proteins	UCH-L1, GFAP
Ranga 2016 (83)	India	Cross sectional study	IS vs. Controls	Not stated	CT; MRI	serum	24h	proteins	CEA
Rahmati 2020 (84)	Iran	Case control study	IS vs. Controls	Neurolo gy ward	CT; MRI	serum	12h	transcripts; proteins	S100B, miR-602

Qi 2021 (85)	China	Case control study	IS vs. Controls	Not stated	CT; MRI	serum; extracellular vesicle (EV)-derived	6h	transcripts	miR-124-3p
Peycheva 2021 (86)	Bulgaria	Cross-sectional study	IS vs. Controls	Neurology ward	CT	serum	24h	proteins	fibrinogen
Perovic 2017 (87)	Croatia	Case control study	IS vs. Controls	Neurology ward	CT	serum	24h	proteins	resistin, copeptin
Penn 2018 (88)	Canada	Prospective study	IS vs. Controls	ED	CT; MRI	plasma	24h	Proteomics	E-selectin, Apolipoprotein C-I, Calponin, Coagulation factor XII, Clusterin, CRP, IGF-1, Complement component 4b (C4b and C4a), Serum paraoxonase/aryl esterase 1(Paraoxonase- PON1), Prothrombin/thrombin, Plasminogen/plasmin/angiostatin, Vitamin K-dependent protein S (Protein S), Serum paraoxonase/lactonase 3 (Paraoxonase- PON3), Vitamin K-dependent protein C (Protein C), Antithrombin III, Vitamin K-dependent protein Z (Protein Z), Coagulation factor V, Apolipoprotein D, Coagulation factor XI, Insulin-like growth factor-binding protein 3 (IBP 3), L-selectin, Plasma protease C1 inhibitor (C1 inhibitor), Plasma serine protease inhibitor (Protein C inhibitor), IL-6, S100A12, Fatty acid binding protein 3 (FABP3), Guanylate cyclase A(NPR1) (ANPR1), Epidermal growth factor receptor (EGFR), Platelet endothelial cell adhesion molecule (PECAM 1), Prolactin
Park 2013 (89)	Korea	Prospective study	IS vs. SM	Not stated	CT; MRI	plasma	24h	proteins	H-FABP, S100B
Park 2018 (90)	USA	Prospective study	IS vs. Controls	Not stated	CT; MRI	plasma	12h	proteins	GPBB
Pan 2020 (91)	China	Case control study	IS vs. Controls	Not stated	Not stated	platelet	7.5h	Genomics	EGR2 gene
Palm 2018 (92)	Germany	Case control study	IS vs. Controls	Not stated	CT; MRI	serum	24h	proteins	MMP-8, MPO, TIMP-1
Oraby 2019 (93)	Egypt	Case control study	IS vs. Controls	Neurology ward	CT; MRI	serum	24h	proteins	thioredoxin
O'Connell 2019 (94)	USA	Case control study	IS vs. SM	ED	CT; MRI	whole blood	24h	genes; leukocyte count	PLXDC2 gene, STK3 gene, ANTXR2 gene, KIF1B gene, CD163 gene, PDK4 gene, CTSZ gene, GRAP gene, MAL gene, ID3 gene
O'Connell 2019 (95)	USA	Case control study	IS vs. HS; Total stroke vs. SM ; Other: HS vs. IS+SM	ED	CT; MRI	whole blood	12h	Proteomics	NVAVAQDENLAG, NNYWANVASGLG, QSLKPKGVALSG, GASVHDGVALSG, GEYFRWNWDSVA,APFGQKDVALGL, GDRRPLGVALSG,KGQRGYHLKHDA, AEQREFNKHLSA,PEFRELSKHDVA, PKPHGFPGQEYV,KPEKLNGVALSG, NSLKENGVALSG,VLGPRHEPDSGA, EKLYYHDSQEKH,AWQKSKGVALSG, QRPDPKDGQAKD
O'Connell 2016 (96)	USA	Case control study	IS vs. SM; IS vs. Controls	ED	MRI	whole blood	4.5h; 5.3h	Genomics	ANTXR2 gene, STK3 gene, PDK4 gene, CD163 gene, MAL gene, GRAP gene, ID3 gene, CTSZ gene, KIF1B gene, PLXDC2 gene
O'Connell 2017 (97)	USA	Case control study	IS vs. SM	ED	CT; MRI	plasma	4.5h	cell free DNA	cfDNA

O'Connell 2017 (98)	USA	Case control study	IS vs. Controls	public platform	Not stated	whole blood	24h; 5h; 3h	genes	ANTXR2 gene, STK3 gene, PDK4 gene, CD163 gene, MAL gene, GRAP gene, ID3 gene, CTSZ gene, KIF1B gene, PLXDC2 gene
O'Connell 2020 (99)	USA	Case control study	IS vs. Controls	ED	CT; MRI	plasma	24h	proteins	NfL, Tau
Nielsen 2020 (100)	Denmark	Prospective study	IS vs. TIA; IS vs. Controls	Neurology ward	CT; MRI	plasma	8h	proteins	NfL, VEGF-A, VCAM-1, ICAM-1, IL-6, S100B, E-selectin
Nguyen 2020 (101)	Netherland	Prospective study	IS vs. HS; IS vs. SM; IS vs. Controls; HS vs. SM	ED	CT	plasma	6h	Transcriptomics	tRNA-TyrGTA, tRNA-ThrCGT, tRNA-ValCAC
Nahan 2017 (102)	USA	Case control study	IS vs. HS; IS vs. Controls; HS vs. Controls	ED	Not stated	plasma	12h	Proteomics	IS vs. control (As, Co, Fe, Li, Sr, U, Se, Cd), HS vs. control (Ag, Al, As , Co, Ni, U , Zn, Fe, Sr, Cd, Pb, Se); HS vs. IS (Ag, Co, Fe, Al, As, Li, Ni, U, W), IS special markers (calpain-15, titin Isoform 3, tropomyosin alpha-4 chain); HS special markers (bestrophin-3, GIRK-1, TTBK1, CAB3)
Montaner 2012 (103)	Spain	Prospective study	IS vs. HS	ED	CT	plasma	6h	proteins	S100B, sRAGE
Menon 2018 (104)	India	Prospective study	IS vs. Controls	Not stated	Not stated	serum	24h; 1h	proteins	IMA
Mattila 2021 (105)	Finland	Prospective study	IS vs. HS	ED	Not stated	plasma	3h; 1h	proteins	GFAP
Matsuo 2013 (106)	Japan	Prospective study	IS vs. Controls	Not stated	CT; MRI	plasma	24h	proteins	VEGF
Matsumori 2002 (107)	Japan	Case control study	IS vs. Controls	Not stated	CT	serum	24h	proteins	HGF
Maly 2021 (108)	Czech Republic	Cross-sectional study	IS vs. Controls	Not stated	Not stated	plasma	4.5h	Metabolomics; lipidomics	FA (20:2), FA (20:3), FA (20:4), FA (20:5), FA (22:4), FA (22:5), FA (22:6), FA (16:1), FA (17:1), AHFA (14:0/16:2), FAHFA (16:1/18:3), FAHFA (18:1/20:3), FAHFA (18:2/20:4), FAHFA (20:4/18:3), LPC (20:5), LPC (22:5), LPC (22:6), LPE (18:2), LPE (20:4), LPE (22:6), LPI (18:1), LPI (18:2), TG (14.0_16.1_20.3), TG (16:0_16:1_18:0), TG (16:0_18:2_18:3), TG (16:0_18:2_22:6), TG (17:1_17:2_19:0)
Mahovic 2013 (109)	Croatia	Prospective study	IS vs. Controls	Not stated	CT	serum	24h	proteins	soluble Fas/APO 1 (sFas/APO 1)
Ma 2019 (110)	China	Case control study	IS vs. Controls	ED + Neurology department	CT; MRI	plasma	6h	transcripts	miR-93
Luger 2017 (111)	Germany	Retrospective study	IS vs. HS; Other: HS vs. IS+SM	ED + Neurology department	CT; MRI	serum	6h	proteins	GFAP
Lu 2020 (112)	China	Case control study +	IS vs. Controls	Not stated	MRI	whole blood	24h; 3h	Transcriptomics	circ-PHKA2, circ-BBS2

		animal experiment study							
Long 2013 (113)	China	Cross-sectional study	IS vs. Controls	Not stated	Not stated	plasma	24h	transcripts	miR-30a, miR-126, let-7b
Llombart 2016 (114)	Spain	Retrospective study	IS vs. HS	ED	CT	plasma	6h	proteins	RBP4, GFAP
Liu 2015 (115)	China	Prospective study	IS vs. Controls	Neurology ward	MRI	serum	24h	transcripts; proteins	miR-124, miR-9, miR-219, MMP9
Liu 2017 (116)	China	Case control study	IS vs. Controls	Not stated	CT; MRI	serum	9h	Metabolomics	serine, isoleucine, betaine, PC (5:0/5:0), LysoPE (18:2)
Liu 2020 (117)	China	Case control study	IS vs. HS; IS vs. Controls	Neurology ward	CT; MRI	serum	24h	proteins	Sphingosine 1-phosphate (S1P)
Liswati 2009 (118)	Indonesian	Case control study	IS vs. HS	Not stated	CT	plasma	24h	proteins	S100B, MBP
Li 2021 (119)	China	Case control study	IS vs. Controls	Neurology ward	MRI	serum	24h	proteins	Lp-PLA2
Li 2015 (120)	China	Case control study	IS vs. Controls	Not stated	CT; MRI	serum	24h	Transcriptomics	miR-32-3p, miR-106-5p, miR-532-5p, miR-1246
Li 2018 (121)	China	Prospective study	IS vs. Controls	ED	CT; MRI	plasma; lymphocytes, neutrophils	6h	transcripts; proteins	miR-424, TNFa, IGF1
Leung 2014 (122)	China	Prospective study	IS vs. HS; IS vs. Controls; HS vs. Controls	ED	CT; MRI	plasma	24h; 6h	transcripts	miR-124-3p, miR-16
Laterza 2006 (123)	USA	Case control study + animal experiment study	IS vs. Controls	Not stated	Not stated	plasma	24h	Genomics; proteins	VLP-1 gene, VLP-1
Laskowitz 2009 (124)	USA	Prospective study	IS vs. HS; Total stroke vs. SM; Total stroke vs. TIA	Not stated	CT; MRI	serum	24h; 12h; 6h; 3h	proteins	MMP9, BNP, D-dimer, S100B
Kokocinska 2007 (125)	Poland	Case control study	IS vs. Controls	Neurology ward	CT	plasma	24h	proteins	S100B, Tissue Polypeptide Antigen (TPA)
Kokocinska 2005 (126)	Poland	Case control study	IS vs. Controls	Neurology ward	CT	serum	12h	proteins	S100B
Kodali 2013 (127)	USA	Case control study	IS vs. HS; IS vs. SM; HS vs. SM	ED	Not stated	plasma	12h	Proteomics	Fibrinogen gamma chain, Protein kinase C eta type, Fibrinogen beta chain, Fibrinogen alpha chain, Complement C3, Methylenetetrahydrofolate reductase, Antithrombin-III, Collagen alpha-1(IV) chain
Kodali 2012 (128)	USA	Case control study	IS vs. HS; IS vs. SM; HS vs. SM	ED	Not stated	plasma	12h	Proteomics	metalloproteins: Mg, Mn, Cu, Se, Pb, Mo
Kochanowski 2012 (129)	Poland	Case control study	IS vs. Controls	Neurology ward	CT	plasma	24h	proteins	resistin, TNFa
Kavalci 2011 (130)	Turkey	Prospective study	IS vs. HS	ED	Not stated	serum	24h	proteins	BNP, D-dimer, MMP-9, S100B
Katsanos 2017 (131)	Greece	Prospective study	IS vs. HS; HS vs. SM; HS vs. Controls	ED	Not stated	plasma	6h	proteins	GFAP

Kashyap 2009 (132)	India	Case control study	IS vs. Controls	Neurology ward	CT	serum	24h	proteins	ITIH4
Kara 2014 (133)	Turkey	Prospective study	IS vs. Controls	ED	MRI	serum	24h	proteins	hsCRP, Lp-PLA2
Kalra 2021 (134)	Germany	Prospective study	IS vs. HS; HS vs. IS+SM	Neurology ward	CT; MRI	serum	12h	proteins	GFAP
Kalani 2020 (135)	USA	Prospective study	IS vs. HS	ED	CT; MRI	plasma	24h	Transcriptomics; extracellular vesicles	miR-150-3p, miR-4286, miR-132-3p, miR-30e-3p, miR-21-3p, miR-27b-3p, miR-342-3p, miR-186-5p, miR-338-3p, miR-5010-5p, miR-134-5p, miR-7c-5p, miR-485-5p
Jin 2017 (136)	China	Case control study	IS vs. Controls	Neurology ward	CT; MRI	plasma	24h	transcripts	miR-126, miR-130a, miR-222, miR-218, miR-185
Jiang 2011 (137)	China	Case control study	IS vs. Controls	Not stated	CT; MRI	serum	6h	Metabolomics	folic acid, cysteine, S-adenosyl-homocysteine, oxidized glutathione, Tetrahydrofolate, Hydroxy eicosatetraenoic acid, Adenosine, Aldosterone, Hydroxy octadecadienoic acid, Deoxocathasterone, Sucrose 6-phosphate, Betanin
Jia 2015 (138)	China	Prospective study	IS vs. Controls	Neurology ward	MRI	serum	24h	transcripts; proteins	miR-145, miR-23a, miR-221, hsCRP, IL-6
Ji 2016 (139)	China	Case control study	IS vs. Controls	Neurology ward	CT; MRI	serum; exosome	24h	transcripts	miR-9, miR-124
Inoue 2019 (140)	Japan	Prospective study	IS vs. HS	Not stated	CT; MRI	serum	24h	proteins	LOX-1
Iltumur 2006 (141)	Turkey	Case control study	IS vs. Controls	NICU	CT; MRI	plasma	24h	proteins	NT-proBNP, troponin I, CK-MB
Herisson 2010 (142)	France	Prospective study	IS vs. HS; Total stroke vs. controls	stroke department	CT; MRI	serum	4.5h	proteins	IMA, HFABP
Han 2012 (143)	China	Case control study	IS vs. Controls; HS vs. Controls	Not stated	CT; MRI	serum	3h	proteins	IMA
Gunduz 2008 (144)	Turkey	Case control study	IS vs. HS; IS vs. Controls; HS vs. Controls	ED	CT; MRI	serum	24h	proteins	IMA
Gunaydin 2014 (145)	Turkey	Prospective study	IS vs. Controls	ED	CT; MRI	plasma	12h; 6h	proteins	SCUBE1
Glickman 2011 (146)	USA	Prospective study	IS vs. SM	ED	CT	plasma	5h	proteins	BNP, MMP-9, D-dimer, S100B, CRP
Giannopoulos 2008 (147)	Greece	Case control study	IS vs. Controls	Not stated	CT	plasma	24h	proteins	Endothelin-1, CRP, fibrinogen
Garlichs 2003 (148)	Germany	Case control study	IS vs. TIA; IS vs. Controls; TIA vs. Controls	Neurology ward	CT	serum; plasma	24h	proteins	platelet CD154, platelet P-selectin, soluble CD154, monocyte CD40, MCP-1
Foerch 2012 (149)	Germany	Prospective study	IS vs. HS; IS vs. SM; HS vs. SM	Stroke centre	CT; MRI	plasma	4.5h	proteins	GFAP
Foerch 2006 (150)	Germany	Prospective study	IS vs. HS	Stroke unit or NICU	CT; MRI	serum	6h	proteins	GFAP
Fiszer 1998 (151)	Poland	Case control study	IS vs. Controls	Not stated	CT	whole blood	12h	proteins	CD54, CD11a, CD11b, CD18
Fassbender 1997 (152)	Germany	Case control study	IS vs. Controls	Not stated	CT	serum	24h; 10h, 8h, 4h	proteins	S100B, NSE

Fang 2018 (153)	China	Prospective study	IS vs. HS; IS vs. SM; IS vs. Controls; HS vs. SM; HS vs. Controls; Total stroke vs. controls	ED	Not stated	plasma	24h	proteins	S100B, CRP, IL-6, PAI-1, MMP-9, P-selectin, ICAM-1, TNFa, LDL, IL-10, NO, GFAP
Ewida 2021 (154)	Egypt	Prospective study	IS vs. HS; IS vs. Controls; HS vs. Controls; Total stroke vs. controls	Neurology ward	CT; MRI	serum	24h	transcripts; proteins	IS vs. HS(lncRNAs HIF1A-AS2, lncRNAs LINK-A, mRNA HIF1-α, MDA, VEGF), IS vs. control (PI3K, p?Akt, VEGFR2, TIE2), HS vs. control (PI3K, p?Akt, VEGFR2, TIE2), total stroke vs. control(lncRNAs HIF1A-AS2, lncRNAs LINK-A, mRNA HIF1-?, TAC, VEGF, ANG1, BDNF, PI3K, p?Akt, VEGFR2, TIE2)
Ekingen 2017 (155)	Turkey	Prospective study	IS vs. Controls	ED	CT	serum	24h	proteins	Galectin-3, GFAP
Dvorak 2009 (156)	Germany	Prospective study	IS vs. HS	Not stated	CT; MRI	serum	6h; 4h; 3h; 2h	proteins	GFAP
Duan 2015 (157)	China	Prospective study	IS vs. Controls	ED	CT; MRI	serum	24h	proteins	CXCL12
De Marchis 2018 (158)	Switzerland	Prospective study	IS vs. TIA; IS+TIA vs. control	ED	MRI	serum	24h	proteins	NfL
Dassan 2012 (159)	UK	Prospective study	IS vs. SM	ED	MRI	serum	24h	proteins	VEGF
Dambinova 2003 (160)	Russia	Prospective study	IS vs. HS; IS vs. TIA; IS vs. Controls; TIA vs. Controls	Neurology and Neurosurgery Department	CT; MRI	serum	3h	proteins	NR2A/2B aAb
Dambinova 2012 (161)	USA	Prospective study	IS vs. Controls	ED	CT; MRI	plasma	12h	proteins	NR2 peptide
Cheng 2018 (162)	China	Prospective study	IS vs. Controls	ED	CT; MRI	serum	24h	transcripts	miR-148b-3p, miR-151b, miR-27b-3p
Chen 2018 (163)	China	Prospective study	IS vs. Controls	Not stated	MRI	serum; plasma	24h	transcripts; proteins	miR-146b, hsCRP, IL-6
Cavrak 2021 (164)	USA	Case control study	IS vs. TIA; IS vs. SM; TIA vs. SM	ED	MRI	whole blood	24h	cell count and percentage	neutrophil percentage > 60
Cano 2003 (165)	Venezuela	Prospective study	IS vs. Controls	ED	CT	serum	24h	metabolites	malondialdehyde, nitric oxide
Cakmak 2014 (166)	Turkey	Prospective study	IS vs. Controls	ED	CT; MRI	serum	24h	proteins	IMA, S100B, NSE
Büttner 1997 (167)	Germany	Prospective study	IS vs. Controls	Not stated	CT	serum	24h; 12h	proteins	S100B
Bustamante 2021 (168)	Spain	Prospective study	IS vs. HS	ED	CT; MRI	plasma	4.5h	proteins	GFAP, RBP-4, NT-proBNP, endostatin
Bustamante 2017 (169)	Spain	Prospective study	IS vs. HS; Total stroke vs. SM	ED	CT; MRI	plasma	6h	proteins	NT-proBNP, IGFBP-3, TNF-R1, GroA, FasL, IL-6, D-dimer, vWF, VAP-1, Endostatin, S100B, Hsc70, Apo CIII, NCAM, MMP-9, bNGF, Caspase-3, NSE, cFn, IL-2RG, IL-17A

Bolayir 2019 (170)	Turkey	Prospective study	IS vs. Controls	Neurology ward	CT; MRI	serum	24h	proteins	SCUBE1, hsCRP
Bibl 2012 (171)	Germany	Case control study	IS vs. Controls	Not stated	CT; MRI	plasma	12h	proteins	Abeta1-37, Abeta1-38
Barr 2010 (172)	UK	Case control study	IS vs. Controls	Not stated	MRI	whole blood	24h	Genomics	ARG1 gene, CA4 gene, CCR7 gene, CSPG2 gene, IQGAP1 gene, LY96 gene, MMP9 gene, ORM1 gene, S100A12 gene
Azarpazhooh 2010 (173)	Iran	Case control study	IS vs. HS; Total stroke vs. controls	Not stated	CT; MRI	serum	24h	proteins	anti-HSP27, hsCRP
Atik 2016 (174)	Turkey	Prospective study	IS vs. Controls; HS vs. Controls	ED	CT; MRI	serum	12h; 3h	proteins	albumin, ischemic modified albumin (IMA), IMA/albumin ratio (IMAR), total antioxidant status, total oxidant status (TOS), oxidative stress index (OSI)
Alvarez-Perez 2011 (175)	Portugal	Prospective study	IS vs. Controls	ED	Not stated	plasma	24h	proteins	fibrinogen, CRP
Allard 2004 (176)	Switzerland	Prospective study	IS vs. HS; IS vs. Controls; HS vs. Controls; Total stroke vs. controls	ED	CT; MRI	plasma	6h	Proteomics	ApoC-I, ApoC-III
Allard 2005 (177)	Switzerland	Retrospective study	IS vs. Controls; HS vs. Controls; TIA vs. Controls; Total stroke vs. controls	ED	CT; MRI	plasma	24h	proteins	PARK7, NDKA
Algin 2019 (178)	Turkey	Prospective study	IS vs. Controls	ED	MRI	serum	4h	proteins	BDNF, VILIP-1
Algawwam 2021 (179)	Iraq	Prospective study	IS vs. Controls	Not stated	Not stated	serum	24h	proteins	GPBB
Ahn 2011 (180)	Korea	Prospective study	IS vs. SM	ED	CT; MRI	serum	6h	proteins	IMA index, IMA
Sadik 2021 (181)	Egypt	Prospective study	IS vs. Controls	Not stated	CT	serum	12h	transcripts	miR-155, JAK2 mRNA, STAT3 mRNA
Abe 2020 (182)	Japan	Prospective study	IS vs. Controls	Not stated	Not stated	whole blood	12h	Transcriptomics	miR-505-5p, miR-1255b-5p, miR-550b-2-5p, miR-4523, miR-6795-3p

IS: ischemic stroke; HS: haemorrhagic stroke; SM: stroke mimics; TIA: transient ischemic attack

Supplementary Table 3: Brain-specific or enriched biomarkers for diagnosis of ischaemic stroke in early stage

**Brain-specific/enriched biomarkers**	**Study ID**	**Study design**	**Sample size**	**Specimen**	**Time point**	**Comparison**	**Sensitivity**	**Specificity**	**AUC**
**BDNF**	Algin 2019 (178)	Prospective	IS 75, control (healthy) 28	**serum**	4h		100%	92%	0.983(0.966-1)
**VILIP-1**							NA	NA	NA
**B-FABP**	Wunderlich 2005(21)	Prospective	IS 42, control (healthy) 80	**serum**	3,4,5,6,12,18,24h		NA	NA	NA
**MBP**	Can 2015 (27)	Prospective	IS 50, control (healthy) 34	**serum**	12h		NA	NA	NA
**miR-107**	Yang 2016 (46)	Case control	IS 114, control (healthy) 58	**plasma**	24h		93.8%	92.2%	0.97
**miR-128b**							72.8%	92%	0.903
**miR-153**							91.2%	74%	0.893
**miR-124**	Zhou 2021 (1)	Case control	IS 108, control (healthy) 108	**serum**	24h		91.67%	93.52%	0.9527
	Liu 2015 (115)	Prospective	IS 31, control (healthy) 11	**serum**	24h				0.76(0.61-0.92)
**GMFB**	Yuan 2020 (44)	Case control	IS 40, control (healthy) 40	**plasma**	24h		NA	NA	NA
**miR-134**	Zhou 2018 (36)	Case control	IS 50, control (healthy) 50	**serum; exosome**	24h		75.3%	72.8%	0.834(0.88-0.97)
**miR-16**	Tian 2016 (62)	Prospective	test cohort: IS 7, control(healthy) 4; validation cohort: IS 33, control (healthy) 23	**plasma**	6h		69.7%	87%	0.775
**miR-9**	Ji 2016 (139)	Case control	IS 65, control (non-stroke) 66	**serum; exosome**	24h	**IS vs. control**	NA	NA	0.8026(0.7235-0.8816)
**miR-124**							NA	NA	0.6976(0.6506-0.7895)
**NfL**	Tiedt 2018 (32)	Prospective	IS 196, control (healthy) 30; cohort2: 95 IS	**serum**	24h		NA	NA	NA
**NfL**	O'Connell 2020 (99)	Case control	IS 14, control (risk factor non-neurological symptom) 33	**plasma**	24h		92.9%	84.9%	0.88(0.75-0.96)
**Tau**							85.7%	54.6%	0.69(0.54-0.82)
**NfL+ Tau**							71.4%	87.9%	0.81(0.67-0.91)
**NfL**	Nielsen 2020 (100)	Prospective	IS 31, TIA 9, control (healthy) 29	**plasma**	8h		NA	NA	NA
**S100B**							NA	NA	NA

**S100B**	Kokocinska 2005 (126)	Case control	IS 47, control (healthy) 10	**serum**	12h		NA	NA	NA
	Büttner 1997 (167)	Prospective	IS 26, control (healthy) 26	**serum**	12h, 24h		NA	NA	NA
	Fang 2018 (153)	Prospective	IS 262, control (healthy) 200	**plasma**	24h		NA	NA	0.98
**S100B**	Fassbender 1997 (152)	Case control	IS 24, control (healthy) 24	**serum**	8h,10h, 24h		NA	NA	NA
**NSE**							NA	NA	NA
**S100B**	Cakmak 2014 (166)	Prospective	IS 38, control (ED patients without acute focal neurological deficit) 30	**serum**	24h		87%	72%	0.89(0.81-0.96)
**NSE**							61%	53%	0.67(0.55-0.80)
**S100B+NSE**							92%	47%	NA
**GFAP**	Ekingen 2017 (155)	Prospective	IS normal CT 17, control (healthy) 50	**serum**	24h		70.59 %	70%	NA
**VILIP-1(VLP-1)**	Stejskal 2011 (64)	Case control	IS 16, control (healthy) 17	**serum**	3h		100%	100%	1.0 (0.93-1)
	Laterza 2006 (123)	Case control	IS 18, control not stated	**plasma**	24h		NA	NA	NA
**GFAP**	Bustamante 2021 (168)	Prospective	IS 154, HS 35, SM 62	**plasma**	4.5h	**IS vs. HS**	37.1%	99.4%	NA
**GFAP**	Stanca 2015 (65)	Prospective	IS 49, HS(ICH) 23, control (healthy) 52	**serum**	12h		94%	69%	NA
**aAbs-NR2+GFAP**							94%	91%	NA
**GFAP**	Xiong 2015(47)	Prospective	IS 65, HS(ICH) 43	**serum**	6h		86%	76.9%	0.901(0.808-0.95)
	Rozanski 2017 (77)	Prospective	IS 49, HS(ICH) 25	**plasma**	1h		24%	100%	NA
					3h		36%	100%	NA
	Mattila 2021 (105)	Prospective	IS 203(IS 195, TIA 8), HS 60 (ICH 50, SAH 2, both 8), SM 9	**plasma**	3h		96.6%	68%	0.781(0.712-0.85)
	Llombart 2016(114)	Retrospective	cohort1: IS 36, HS(ICH)10, cohort2: IS16, HS(ICH)16, cohort3: IS 38, HS(ICH)28	**plasma**	6h		68.4% (GFAP+RBP4)	84% (GFAP+RBP4)	NA
	Katsanos 2017 (131)	Prospective	IS 121, HS 36(ICH 31, SAH 5), SM 31, control (healthy)79	**plasma**	6h		91%	97%	0.97(0.92-1.00)
	Foerch 2012 (149)	Prospective	IS 163, HS 39, SM 3	**plasma**	4.5h		84.2%	96.3%	0.915(0.847-0.982)
	Foerch 2006 (150)	Prospective	HS 42, IS 93	**serum**	6h		79%	98%	NA
		Prospective	IS 45, HS 18		2h		45%	100%	NA

	Dvorak 2009 (156)			serum	3h	HS vs. IS	65%	100%	NA
					4h		70%	100%	NA
					6h		71%	95%	NA
**miR-124-3p**	Leung 2014 (122)	Prospective	IS 74, HS 19, control (healthy) 23	plasma	24h		68.4%	71.2%	0.70(0.59-0.79)
**miR-16**							94.7%	35.1%	0.66(0.55-0.76)
**S100**	Rainer 2007 (31)	Prospective	IS 118, HS 35, image-negative clinical stroke 44	plasma	24h		NA	NA	NA
	Zhou 2016 (35)	Prospective	IS 71, HS(ICH) 46	plasma	6h		95.7%	70.4%	0.903
**S100B+sRAGE**	Montaner 2012 (103)	Prospective	IS 279, HS(ICH) 58	plasma	6h		22.7%	80.2%	NA
**S100B**	Kavalci 2011 (130)	Prospective	IS 71, HS 29, control 20	serum	24h		NA	NA	NA
**S100B**	Liswati 2009 (118)	Case control	IS 25, HS 19	plasma	24h		77.8%	69.2%	0.778(0.582-0.973)
**MBP**							88.9%	69.2%	0.855(0.699-1.000)
**UCH-L1**	Ren 2016 (82)	Case control	ICH 45, IS 79, SAH 5, TIA 3, control (healthy) 57	serum	4.5h		NA	NA	0.64(0.47-0.81)
					24h		NA	NA	0.62(0.51-0.72)
**GFAP**					4.5h		61%	96%	0.86(0.73-0.99)
					24h		67%	91%	0.87(0.80-0.94)
**CTNND2**	Simats 2020 (69)	Case control	IS 71, SM 36	plasma	6h	**IS vs. SM**	NA	NA	0.76 (0.66-0.86)
**S100B**	Glickman 2011 (146)	Prospective	IS 34, SM 29	plasma	5(3.5-8) h		NA	NA	NA
	Park 2013 (89)	Prospective	IS 111, SM 127	plasma	24h		54%	83.5%	0.7
**NfL**	De Marchis 2018 (158)	Prospective	IS 504, TIA 111, control (healthy) 165	serum	24h	**IS vs. TIA**	NA	NA	NA

GFAP: Glial Fibrillary Acidic Protein; B-FABP: Brain-type Fatty Acid-Binding Proteins; MBP: Myelin Basic Protein; S100B: S100 calcium-binding protein B; NSE: Neuron-Specific Enolase; NfL, Neurofilament Light chain; CTNND2: Catenin delta-2; UCH-L1: Ubiquitin Carboxyl-terminal Hydrolase isozyme L1; VILIP-1(VLP-1): Visinin-like protein 1; GMFB: Glial Maturation Factor Beta; IS: ischaemic stroke; HS: haemorrhagic stroke; SM: stroke mimics; TIA: transient ischaemic attack; ICH: intracerebral hemorrhage; SAH: subarachnoid hemorrhage; ED: emergency department.

Supplementary Table 4: Characteristics of included high-throughput omics studies

Study ID	Design	Omics	Assay	Algorithm/method of data dimension-reduce	Cohort 1	Cohort 2	Cohort 3	Total sample size	Sampling time	Specimen	Biomarker NO.	As a Panel
Tang 2006 (30)	Case control study	**Genomics**	Oligonucleotide microarrays	The Prediction Analysis of Microarrays (PAM)	IS 15, control 3	NO	NO	18	24h; 5h; 3h	whole blood (Paxgene RNA tubes)	18	No
Stamova 2010 (66)	Case control study	**Genomics**	Whole genome microarray	The Prediction Analysis of Microarrays (PAM)	IS 70, control: healthy 38, vascular risk 52, myocardial infarction 17	NO	NO	177	24h; 5h; 3h	whole blood	97	Yes
Pan 2020 (91)	Case control study	**Genomics**	RNA sequencing, qRT-PCR	GO and KEGG enrichment to choose inflammation related genes	IS male 8, control (healthy male) 4	NO	NO	12	7.5h	Platelet	1	No
O'Connell 2016 (96)	Case control study	**Genomics**	RNA microarray	Machine-learning (GA/ KNN)	IS 39, control (neurologically asymptomatic) 24	IS 39, control 30, SM 20	NO	152	4.5h; 5.3h	whole blood (Paxgene RNA tubes)	10	Yes
Barr 2010 (172)	Case control study	**Genomics**	RNA gene expression profiling	BeadStudio; GeneSpring	IS 39, control (non-stroke) 25	NO	NO	63	24h	whole blood (Paxgene RNA tubes)	9	No
Laterza 2006 (123)	Case control study + animal experimental study	**Genomics**	Gene array, ELISA	No advanced algorithm	Mouse	NO	IS 18, control not stated	18	24h	Plasma	1	No
Zhang 2017 (41)	Case control study	**Metabolomics**	MS	Artificial neural network (ANN)	IS 103, HS(ICH)103, control(healthy) 65	IS 26, HS(ICH) 25	NO	322	12h	dried blood spot	36	Yes
Tiedt 2020 (60)	Prospective study	**Metabolomics**	UPLC-MS/MS	Random forest	IS 40, SM 33, control (neurological normal) 72	IS 105, SM 105, control 40	IS 289, SM 211	969	24h	Serum	4	Yes

Liu 2017 (116)	Case control study	**Metabolomics**	MS	Logistic regression, Naïve Bayes, Neural network, Random Forest, Support vector machine	IS 40, control 40	IS 26, control 23	NO	129	9h	Serum	5	Yes
Jiang 2011 (137)	Case control study	**Metabolomics**	UPLC-TOF-MS	K-Nearest Neighbour (KNN)	IS 67, control (routine physical check-up people) 62	NO	NO	129	6h	Serum	12	No
Maly 2021(108)	Cross-sectional study	**Metabolomics(lipidomics)**	UHPLC-HRMS	supervised statistical methods	IS 49, control 82	NO	NO	131	4.5h	Plasma	27	No
Kimberly 2013(28)	Prospective study + animal experimental study	**Metabolomics; targeted**	LC-MS/MS	Principal component analysis (PCA), partial least-squares discriminant analysis (PLS-DA)	Rat	IS 32, control (TIA + non-stroke) 52	NO	84	2h; 9h	Plasma	18	Yes
Turek-Jakubowska 2022 (4)	Case control study	**Proteomics**	LC-MS	Diffprot software	IS 32, control 28	NO	NO	60	24h	Plasma	1	No
Song 2019(68)	Case control study + animal experimental study	**Proteomics**	LC-MS/MS, ELISA	Principal component analysis (PCA)	Mouse	IS 70, control (healthy) 70	NO	140	24h	Serum	2	No
Sharma 2015(73)	Case control study	**Proteomics**	iTRAQ-Based LC-MS	None	IS 50, control (healthy) 35	NO	NO	85	24h	Serum	3	No
Rico Santana 2014(79)	Case control study	**Proteomics**	MALDI-TOF MS	None	IS 45, control (TIA+ HS+ SM+ head injury) 18, control (healthy) 56	NO	NO	119	6h	Serum	1	No
Penn 2018 (88)	Prospective study	**Proteomics; targeted**	LC/MRM-MS, ELISA	None	IS 20, control (outpatient neurology clinic) 20	NO	NO	40	24h	Plasma	30	Yes
O'Connell 2019 (95)	Case control study	**Proteomics**	Custom-fabricated high-density peptide array	Random forest	IS 19, HS 17, SM 20	NO	NO	56	12h	whole blood	17	Yes
Nahan 2017 (102)	Case control study	**Proteomics（targeted metalloprotein）**	MS	Principal component analysis (PCA)	IS 14, paired control 14; HS(ICH) 23, paired control 23	NO	NO	74	12h	Plasma	36	No

Kodali 2013 (127)	Case control study	**Proteomics; targeted**	Nano-LC-ESIMS	None	IS 10, HS 9, SM 10	NO	NO	29	12h	Plasma	8	No
Kodali 2012 (128)	Case control study	**Proteomics (metals and metalloproteins)**	ICP-MS followed by ESIMS	None	IS 10, HS 9, SM 10	NO	NO	29	12h	Plasma	6	No
Gawryś 2022 (9)	Case control study	**Proteomics (platelet)**	LC-MS	None	IS 32, control 29	NO	NO	61	24h	platelet	8	No
Simats 2018 (70)	Case control study + animal experimental study	**Proteomics**	SOMAscan proteomic assay, ELISA	None	Rat	IS 38, control 8	NO	46	6h	Plasma	2	No
Yang 2022 (3)	Case control study	**Transcriptomics**	CircRNA microarray, qRT-PCR	Volcano plot and heatmap	IS 10, control 10	NO	NO	20	12h	plasma; exosome	4	No
Tian 2022 (5)	Case control study	**Transcriptomics**	RNA microarray, qRT-PCR	Heatmap and hierarchical clustering	IS 39, control 39	NO	NO	78	6h	whole blood	1	No
Tiedt 2017 (59)	Prospective study	**Transcriptomics**	RNA Sequencing	None	IS 20, control (healthy) 20	IS 40, control (healthy) 40	IS 200, control (healthy) 100, TIA 72	492	24h	Plasma	3	Yes
Tian 2016 (62)	Prospective study	**Transcriptomics**	miRNAs microarrays, qRT-PCR	Clustering heatmap	IS 7, control (healthy) 4	IS 33, control (healthy) 23	NO	67	6h	Plasma	1	No
Stamova 2019 (67)	Case control study	**Transcriptomics**	RNA microarray	None	IS 33, HS (ICH) 33, control (risk factors) 33	NO	NO	99	24h	not stated	not exactly	No
Sepramaniam 2014 (75)	Prospective study + animal experimental study	**Transcriptomics**	microRNA microarray, qRT-PCR	None	IS 68, control (healthy) 24	IS 101	Metabolic syndrome patients 94<br><br>At last, validation in rats	287	24h	whole blood	5	No
Nguyen 2020 (101)	Prospective study	**Transcriptomics**	Small RNA-sequencing	LASSO (least absolute shrinkage and selection operator)	IS 9, HS(ICH)8, SM 9	IS 20, control (healthy) 20	NO	66	6h	Plasma	3	Yes
Lu 2020 (112)	Case control study + animal	**Transcriptomics**	CircRNA Microarray, RT-qPCR	None	Mouse		NO	16	24h; 3h	whole blood	2	No

	experimental study					IS 8, control 8						
Li 2015 (120)	Case control study	**Transcriptomics**	miRNA microarray, qRT-PCR	None	IS 117, control (healthy) 82	IS 53, control 50	NO	199	24h	Serum	4	No
Abe 2020 (182)	Prospective study	**Transcriptomics**	miRNA Array	Principal component analysis (PCA)	IS 22, controls (without stroke symptoms) 22	NO	NO	44	12h	whole blood	5	No
Kalani 2020 (135)	Prospective study	**Transcriptomics**	RNA sequencing	LASSO (least absolute shrinkage and selection operator)	IS 21, HS 36 (ICH 19, SAH 17)	NO	NO	57	24h	Plasma	13	Yes
Simats 2020 (69)	Case control study + animal experimental study	**Transcriptomics + Proteomics**	Whole-genome microarray, BCA Protein Assay, LC-MS, ELISA	Ingenuity Pathway Analysis (IPA), Principal Component Analysis (PCA), Multiple Co-Inertia Analysis (MCIA), regularized Canonical Correlation Analysis (rCCA), Multiple Factor Analysis	Mouse	IS 71, SM 36	NO	107	6h	Plasma	1	NO

IS: ischemic stroke; HS: haemorrhagic stroke; SM: stroke mimics; TIA: transient ischemic attack

Supplementary list 5: Biomarkers pooled from high-throughput omics studies

103 Genes:

ABCA1, AMN1, ANKRD28, ANTXR2, ARG1, ASTN2, BCL6, BST1, RPF1, CA4, CCDC144A, CCR7, ACKR4, CD163, CHMP1B, CKAP4, CKLF, CLEC4E, CPEB2, CSPG2, CTSZ, CXADR, DLX6, EGR2, ELAVL2, ELL2, ENTPD1, ETS2,F5, FAR2, FAT3, FCHO2, FCRL4, FGD4,FOXA2, FPR1, GAB1, GABRB2, GADL1, GLYATL1, GRAP, GYPA, H2AC18, HNRNPH2, HNRNPUL2, ID3, IFRD1, IQGAP1, KIAA0319, KIF1B, LAMP2, LDB3, LECT2, PPFIBP1, LTB4R, LY96, MAL, MCTP1, MMP9, NPL, ORM1, OSBPL1A, OVOL2, PDK4, PGM5, PHTF1, PLXDC2, PRTG, PTPRD, PYGL, QKI, RBM25, RBMS3, REM2, RNASE2, RNF141, RPL22, RRAGD, S100A12, S100A9, S100P, SCD5, SH3GL3, SHOX, SLC16A6, SNIP1, SNRPN, SPIB, SPTLC3, ST3GAL6, STK3, TBX5, THBD, THSD4, TIMM8A, TIMP2, UBR5, UBXN2B, UNC5B, VAPA, VSNL1, VNN3, ZNF608

79 proteins (or peptides):

2155-Da peptide, ADAMTS13, AEQREFNKHLSA, Alpha-1B-glycoprotein, Amyloid-like protein 2, Antithrombin-III, APFGQKDVALGL, ApoC-I, ApoC-III, Apolipoprotein D, AWQKSKGVALSG, Beta-amyloid protein A4, calpain-15, Calponin, Coagulation factor XII, CKB, Clusterin, CMPK, coactosin-like protein, Coagulation factor V, Coagulation factor XI, Collagen alpha-1(IV) chain, Complement C3, Complement component 4b (C4b and C4a), CRP, CTNND2, cystatin C (CST3),E-selectin, EKLYYHDSQEKH, Epidermal growth factor receptor (EGFR), Fatty acid binding protein 3 (FABP3),Fibrinogen alpha chain, Fibrinogen beta chain, Fibrinogen gamma chain, GASVHDGVALSG, GDRRPLGVALSG, GEYFRWNWDSVA, Guanylate cyclase A(NPR1) (ANPR1), histone proteins (H2A type 1 and 1-A, H2A types 2B and J, H2Av, -z, and -x), IGF-1, IL-6, Insulin-like growth factor-binding protein 3 (IBP 3), interferon regulatory factor 7 (IRF7), KGQRGYHLKHDA, KPEKLNGVALSG, L-selectin, metalloproteins Cu, metalloproteins Mg, metalloproteins Mn, metalloproteins Mo, metalloproteins Pb, metalloproteins Se, Methylenetetrahydrofolate reductase, NNYWANVASGLG, NSLKENGVALSG, NVAVAQDENLAG, PEFRELSKHDVA, PKPHGFPGQEYV, Plasma protease C1 inhibitor (C1 inhibitor), Plasma serine protease inhibitor (Protein C inhibitor), Plasminogen/plasmin/angiostatin, platelet basic protein, Platelet endothelial cell adhesion molecule (PECAM 1), Prolactin, Protein kinase C eta type, Prothrombin/thrombin, QRPDPKDGQAKD, QSLKPKGVALSG, S100A12 (CGRP), S100A7, Serum paraoxonase/aryl esterase 1 (Paraoxonase- PON1), Serum paraoxonase/lactonase 3 (Paraoxonase- PON3), thymidine phosphorylase 4 (TYMP-4), titin Isoform 3, tropomyosin alpha-4 chain, Vitamin K-dependent protein C (Protein C), Vitamin K-dependent protein S (Protein S), Vitamin K-dependent protein Z (Protein Z), VLGPRHEPDSGA, vWF

97 metabolites:

(0+2+3+16+18:1)/Cit, (0+2+3016+18:1)/Cit, 5-adenosylhomocysteine, Adenosine, Aldosterone, Arg, Asparagine, asymmetrical dimethylarginine (ADMA), BCAA isoleucine, BCAA leucine, BCAA valine, Betanin, C10, C10:2, C12, C14, C14:2, C16, C16-OH, C16-OH/C16, C16:1-OH, C18, C18-OH, C18:1, C22, C3/Met, C3DC, C3DC/C10, C4, C4-OH, C4DC, C5, C5/C3, C5DC, C5DC/C16, C5DC/C5-OH, Carnitine, cis/trans hydroxyproline, creatinine, cysteamine, cysteine, Deoxocathasterone, FA (16:1), FA (17:1), FA (20:2), FA (20:3), FA (20:4), FA (20:5), FA (22:4), FA (22:5), FA (22:6), FAHFA (14:0/16:2), FAHFA (16:1/18:3), FAHFA (18:1/20:3), FAHFA (18:2/20:4), FAHFA (20:4/18:3), folic acid, glucose, glycine, hexadecanedioate, histidine, Hydroxy eicosatetraenoic acid, Hydroxy octadecadienoic acid, isoleucine, betaine, LPC (20:5), LPC (22:5), LPC (22:6), LPE (18:2), LPE (20:4), LPE (22:6), LPI (18:1), LPI (18:2), Lysine, LysoPE (18:2), Methionine, N-carbamoyl-beta-alanine, oxidized glutathione, PC (5:0/5:0), pregnenolone sulphate, Pro, Proline, S-adenosyl-homocysteine, Serine, Sucrose 6-phosphate, symmetrical dimethylarginine (SDMA), tetradecanedioate, Tetrahydrofolate, TG (14.0_16.1_20.3), TG (16:0_16:1_18:0), TG (16:0_18:2_18:3), TG (16:0_18:2_22:6), TG (17:1_17:2_19:0), Thr, Threonine, Tyr/Cit, Uridine, Val/Phe

41 transcripts:

circ-BBS2, circ-PHKA2, circ_0041685, circ_0066867, circ_0093708, circ_0112036, lncRNA NR_120420, miR-106-5p, miR-1246, miR-1255b-5p, miR-125a-5p, miR-125b-2*, miR-125b-5p, miR-132-3p, miR-134-5p, miR-143-3p, miR-150-3p, miR-16, miR-186-5p, miR-21-3p, miR-27a*, miR-27b-3p, miR-30e-3p, miR-32-3p, miR-338-3p, miR-342-3p, miR-422a, miR-4286, miR-4523, miR-485-5p, miR-488, miR-5010-5p, miR-505-5p, miR-532-5p, miR-550b-2-5p, miR-627, miR-6795-3p, miR-7c-5p, tRNA-ThrCGT, tRNA-TyrGTA, tRNA-ValCAC

Supplementary Table 6: Quality assessment results using QUADAS-2

STUDY ID	Risk of bias				Concerns of applicability		
	PATIENT SELECTION	INDEX TEST	REFERENCE STANDARD	FLOW AND TIMING	PATIENT SELECTION	INDEX TEST	REFERENCE STANDARD
Zhou 2021 (1)	Unclear	Low	Low	Low	Low	Low	Low
Zhang 2022 (2)	Unclear	High	Unclear	Unclear	Low	Low	Unclear
Yang 2022 (3)	Unclear	Low	Unclear	Unclear	Unclear	Low	Unclear
Turek-Jakubowska 2022 (4)	Unclear	Low	Low	Low	Low	Low	Low
Tian 2022 (5)	Unclear	Low	Unclear	Unclear	Low	Low	Unclear
Rahmati 2021 (6)	Low	Low	Low	Low	Low	Low	Low
Li 2021 (7)	Unclear	Low	Low	Low	Low	Low	Low
Induruwa 2022 (8)	Low	Low	Unclear	Unclear	Low	Low	Unclear
Gawryś 2022 (9)	Low	Low	Low	Low	Low	Low	Low
Cho 2022 (10)	Unclear	Low	Unclear	Unclear	Low	Low	Low
Intiso 2004 (11)	Low	Low	Low	Low	Low	Low	Low
Perini 2001(12)	Low	Low	Low	Low	Low	Low	Low
Pedersen 2004 (13)	Unclear	Low	Low	Low	Low	Low	Low
Nayak 2011 (14)	Unclear	Low	Low	Low	Low	Low	Low
Senes 2007 (15)	Low	Low	Unclear	Unclear	Low	Low	Unclear
Feng 2019 (16)	High	Low	Low	Low	Low	Low	Low
Blann 1999 (17)	Low	Low	Unclear	Low	Low	Low	Unclear
Shyu 1997 (18)	Low	Low	Low	Low	Low	Low	Low
Liu 2015 (19)	Low	Low	Low	Low	Low	Low	Low
Supanc 2011 (20)	Low	Low	Low	Low	Low	Low	Low
Wunderlich 2005(21)	Low	Low	Low	Low	Low	Low	Low
Hu 2016 (22)	High	Low	Unclear	Unclear	Unclear	Low	Unclear

Uno 2003 (23)	Low	Low	Low	Low	Low	Low	Low
Sun 2019 (24)	Unclear	Low	Low	Low	Low	Low	Low
Song 2006 (25)	Low	Low	Low	Low	Low	Low	Low
Zitnanova 2016 (26)	Low	Low	Low	Low	Low	Low	Low
Can 2015 (27)	Low	Low	Low	Low	Low	Low	Low
Kimberly 2013 (28)	Low	Low	Low	Low	Low	Low	Low
Abboud 2007 (29)	Low	Low	Low	Low	Low	Low	Low
Tang 2006 (30)	Unclear	Low	High	Low	Low	Low	Low
Rainer 2007 (31)	Low	Low	Low	Low	Low	Low	Low
Tiedt 2018 (32)	Low	Low	Low	Low	Low	Low	Low
Zhu 2019 (33)	Low	Low	Low	Low	Low	Low	Low
Zhu 2018 (34)	Unclear	Low	Low	Low	Low	Low	Low
Zhou 2016 (35)	Unclear	Low	High	Low	Low	Low	Unclear
Zhou 2018 (36)	Unclear	Low	Low	Low	Low	Low	Low
Zhou 2022 (37)	Low	Low	Low	Low	Low	Low	Low
Zhao 2016 (38)	Low	Low	Unclear	Unclear	Low	Low	Unclear
Zhao 2017 (39)	High	Low	Low	Low	Low	Low	Low
Zhao 2016 (40)	Unclear	Low	Low	Low	Low	Low	Low
Zhang 2017 (41)	Unclear	Low	Low	Low	Low	Low	Low
Zhang 2020 (42)	Low	Low	Low	Low	Low	Low	Low
Zaremba 2006 (43)	Unclear	Low	Low	Low	Low	Low	Low
Yuan 2020 (44)	Unclear	Low	Low	Low	Low	Low	Low
Yigit 2017 (45)	Unclear	Low	Unclear	Unclear	Low	Low	Unclear
Yang 2016 (46)	Unclear	Low	Low	Low	Low	Low	Low
Xiong 2015(47)	Low	Low	Low	Low	Unclear	Low	Low
Wu 2020 (48)	Unclear	Low	Low	Low	Low	Low	Low
Williams 2007 (49)	Low	Low	Low	Low	Low	Low	Low
Wang 2017 (50)	Low	Low	Low	Low	Low	Low	Low
Wang 2014 (51)	Low	Low	Low	Low	Low	Low	Low

Wang 2018 (52)	Unclear	Low	Low	Low	Low	Low	Low
Wang 2017 (53)	Unclear	Low	Low	Low	Low	Low	Low
Walsh 2016 (54)	Low	Low	Unclear	Unclear	Low	Low	Unclear
Vukasovic 2006 (55)	Unclear	Low	Low	Low	Low	Low	Low
von Recum 2015 (56)	Low	Low	Unclear	Unclear	Low	Low	Unclear
Unden 2009 (57)	Low	Low	Low	Low	Unclear	Low	Low
Tunç 2018 (58)	Unclear	Low	Low	Low	Low	Low	Low
Tiedt 2017 (59)	Low	Low	Low	Low	Low	Low	Low
Tiedt 2020 (60)	Low	Low	Low	Low	Low	Low	Low
Tian 2015 (61)	Low	Low	Low	Low	Low	Low	Low
Tian 2016 (62)	Low	Low	Unclear	Unclear	Low	Low	Unclear
Taema 2014 (63)	Unclear	Low	High	Low	Low	Low	High
Stejskal 2011 (64)	High	Low	Low	Low	Low	Low	Low
Stanca 2015 (65)	Low	Low	Low	Low	Low	Low	Low
Stamova 2010 (66)	Unclear	Low	Unclear	Unclear	Unclear	Low	Unclear
Stamova 2019 (67)	Unclear	Low	Low	Low	Low	Low	Low
Song 2019 (68)	Unclear	Low	Low	Low	Low	Low	Low
Simats 2020 (69)	Unclear	Low	Unclear	Unclear	Low	Low	Unclear
Simats 2018 (70)	Unclear	Low	Unclear	Unclear	Unclear	Low	Unclear
Simats 2018 (71)	Unclear	Low	Unclear	Unclear	Low	Low	Unclear
Sharma 2014 (72)	Low	Low	Low	Low	Low	Low	Low
Sharma 2015 (73)	Unclear	Low	Low	Low	Low	Low	Low
Shaker 2020 (74)	Unclear	Low	Low	Low	Low	Low	Low
Sepramaniam 2014 (75)	Unclear	Low	Low	Low	Low	Low	Low
Sayan 2016 (76)	Low	Low	Low	Low	Low	Low	Low
Rozanski 2017 (77)	Low	Low	Low	Low	Low	Low	Low
Roudbary 2011 (78)	High	Low	Low	Low	Low	Low	Low
Rico Santana 2014 (79)	Unclear	Low	Low	Low	Low	Low	Low
Richard 2016 (80)	Unclear	Low	Low	Low	Low	Low	Low

Reynolds 2003 (81)	Low	Low	Low	Low	Low	Low	Low
Ren 2016 (82)	High	Low	Low	Low	Low	Low	Low
Ranga 2016 (83)	High	Low	Low	Low	High	Low	Low
Rahmati 2020 (84)	Unclear	Low	Low	Low	Low	Low	Low
Qi 2021 (85)	Unclear	Low	Low	Low	Low	Low	Low
Peycheva 2021 (86)	Low	Low	Low	Low	Low	Low	Low
Perovic 2017 (87)	Low	Low	Low	Low	Low	Low	Low
Penn 2018 (88)	Unclear	Low	Low	Low	Low	Low	Low
Park 2013 (89)	Low	Low	Low	Low	Low	Low	Low
Park 2018 (90)	Low	Low	Low	Low	Low	Low	Low
Pan 2020 (91)	High	Low	Unclear	Low	High	Low	Unclear
Palm 2018 (92)	Low	Low	Low	High	Low	Low	Low
Oraby 2019 (93)	High	Low	Low	Low	Low	Low	Low
O'Connell 2019 (94)	Unclear	Low	Low	Low	Low	Low	Low
O'Connell 2019 (95)	Unclear	Low	Low	Low	Low	Low	Low
O'Connell 2016 (96)	Unclear	Low	Low	Low	Low	Low	Low
O'Connell 2017 (97)	Unclear	Low	Low	Low	Low	Low	Low
O'Connell 2017 (98)	High	Low	Unclear	Unclear	Low	Low	Unclear
O'Connell 2020 (99)	Unclear	Low	Low	Low	Low	Low	Low
Nielsen 2020 (100)	Low	Low	Low	Low	Low	Low	Low
Nguyen 2020 (101)	Low	Low	Low	Low	Low	Low	Low
Nahan 2017 (102)	High	Low	Unclear	Unclear	Low	Low	Unclear
Montaner 2012 (103)	Low	Low	Low	Low	Low	Low	Low
Menon 2018 (104)	Low	Low	Unclear	Unclear	Low	Low	Unclear
Mattila 2021(105)	Low	Low	Unclear	Unclear	Low	Low	Unclear
Matsuo 2013 (106)	Low	Low	Low	Low	Low	Low	Low
Matsumori 2002 (107)	Low	Low	Low	Unclear	Low	Low	Low
Maly 2021 (108)	Low	Low	Unclear	Unclear	Low	Low	Unclear
Mahovic 2013 (109)	High	Low	Low	Low	Low	Low	Low

Ma 2019 (110)	Unclear	Low	Low	Low	Low	Low	Low
Luger 2017(111)	Low	Low	Low	Low	Low	Low	Low
Lu 2020 (112)	High	Low	Low	Low	Low	Low	Low
Long 2013 (113)	High	Low	Unclear	Unclear	Low	Low	Unclear
Llombart 2016 (114)	High	Low	Low	Low	Low	Low	Low
Liu 2015 (115)	Low	Low	Low	Low	Low	Low	Low
Liu 2017 (116)	High	Low	Low	Low	Low	Low	Low
Liu 2020 (117)	High	Low	Low	Low	Low	Low	Low
Liswati 2009 (118)	High	Low	Low	Low	Low	Low	Low
Li 2021 (119)	Unclear	Low	Low	Low	Low	Low	Low
Li 2015 (120)	High	Low	Low	Low	Low	Low	Low
Li 2018 (121)	Low	Low	Low	Low	Low	Low	Low
Leung 2014 (122)	Low	Low	Low	Low	Low	Low	Low
Laterza 2006 (123)	High	Low	Unclear	Unclear	Unclear	Low	Unclear
Laskowitz 2009 (124)	Low	Low	Low	Low	Low	Low	Low
Kokocinska 2007 (125)	High	Low	Low	Low	Low	Low	Low
Kokocinska 2005 (126)	High	Low	Low	Low	Low	Low	Low
Kodali 2013 (127)	High	Low	Unclear	Unclear	Unclear	Low	Unclear
Kodali 2012 (128)	High	Low	Unclear	Unclear	Unclear	Low	Unclear
Kochanowski 2012 (129)	High	Low	Low	Low	High	Low	Low
Kavalci 2011 (130)	Low	Low	Unclear	Unclear	Low	Low	Unclear
Katsanos 2017 (131)	Low	Low	Unclear	Unclear	Low	Low	Unclear
Kashyap 2009 (132)	High	Low	Low	Low	Low	Low	Low
Kara 2014 (133)	Low	Low	Low	Low	Low	Low	Low
Kalra 2021 (134)	Low	Low	Low	Low	Low	Low	Low
Kalani 2020 (135)	Low	Low	Low	Low	Low	Low	Low
Jin 2017 (136)	High	Low	Low	Low	Low	Low	Low
Jiang 2011 (137)	High	Low	Low	Low	Low	Low	Low

Jia 2015 (138)	Low	Low	Low	Low	Low	Low	Low
Ji 2016 (139)	Unclear	Low	Low	Low	Low	Low	Low
Inoue 2019 (140)	Low	Low	Low	Low	Low	Low	Low
Iltumur 2006 (141)	High	Low	Low	Low	Low	Low	Low
Herisson 2010 (142)	Low	Low	Low	Low	Low	Low	Low
Han 2012 (143)	High	Low	Low	Low	Low	Low	Low
Gunduz 2008 (144)	High	Low	Low	Low	Low	Low	Low
Gunaydin 2014 (145)	Low	Low	Low	Low	Low	Low	Low
Glickman 2011 (146)	High	Low	Low	Low	Low	Low	Low
Giannopoulos 2008 (147)	High	Low	Low	Low	Low	Low	Low
Garlichs 2003 (148)	High	Low	Low	Low	Low	Low	Low
Foerch 2012 (149)	Low	Low	Low	Low	Low	Low	Low
Foerch 2006 (150)	Low	Low	Low	Low	Low	Low	Low
Fiszer 1998 (151)	High	Low	Low	Low	Low	Low	Low
Fassbender 1997 (152)	Low	Low	Low	Low	Low	Low	Low
Fang 2018 (153)	Low	Low	Unclear	Unclear	Low	Low	Unclear
Ewida 2021 (154)	Low	Low	Low	Low	Low	Low	Low
Ekingen 2017 (155)	Low	Low	Unclear	Unclear	Low	Low	Unclear
Dvorak 2009 (156)	Low	Low	Low	Low	Unclear	Low	Low
Duan 2015 (157)	Low	Low	Low	Low	Low	Low	Low
De Marchis 2018 (158)	Low	Low	Low	Low	Low	Low	Low
Dassan 2012 (159)	Low	Low	Low	Low	Low	Low	Low
Dambinova 2003 (160)	High	Low	Low	Low	Low	Low	Low
Dambinova 2012 (161)	Low	Low	Low	Low	Low	Low	Low
Cheng 2018 (162)	Low	Low	Low	Low	Low	Low	Low
Chen 2018 (163)	Low	Low	Low	Low	Unclear	Low	Low
Cavrak 2021 (164)	High	High	Low	Low	Low	Low	Low
Cano 2003 (165)	Low	Low	Low	Low	Low	Low	Low

Cakmak 2014 (166)	Low	Low	Low	Low	Low	Low	Low
Büttner 1997 (167)	High	Low	Low	Low	High	Low	Low
Bustamante 2021 (168)	Low	Low	Low	Low	Low	Low	Low
Bustamante 2017 (169)	Low	Low	Low	Low	Low	Low	Low
Bolayir 2019 (170)	Low	Low	Low	Low	Low	Low	Low
Bibl 2012 (171)	Unclear	Low	Low	Low	Low	Low	Low
Barr 2010 (172)	High	Low	Low	Low	Low	Low	Low
Azarpazhooh 2010 (173)	High	Low	Low	Low	Low	Low	Low
Atik 2016 (174)	Low	Low	Low	Low	Low	Low	Low
Alvarez-Perez 2011 (175)	Low	Low	Unclear	Unclear	Low	Low	Unclear
Allard 2004 (176)	Low	Low	Low	Low	Low	Low	Low
Allard 2005 (177)	Low	Low	Low	High	Low	Low	Low
Algin 2019 (178)	Low	Low	Low	Low	Low	Low	Low
Algawwam 2021 (179)	Low	Low	Unclear	Unclear	Low	Low	Unclear
Ahn 2011 (180)	Low	Low	Low	Low	Low	Low	Low
Sadik 2021 (181)	Low	Low	Low	Low	Low	Low	Low
Abe 2020 (182)	Low	Low	Low	Unclear	Low	Low	Unclear

Supplementary Table 7: Characteristics of included 10 studies for diagnostic meta-analysis

Study ID	Study design	Group	Sample size	Age mean± SD/IQR	Sex male (%)	Setting	Reference	Specimen	Assay	Time point	Comparison	HS medium (IQR)	IS medium (IQR)	TP	FP	FN	TN	Sensitivity	Specificity	Cut-off value	Unit
Dvorak 2009(156)	Prospective study	IS 22, HS 17	39	HS 62 (60-70), IS 75 (61- 83)	HS 10 (55.6%), IS 25(55.6%)	NA	CT; MRI	serum	ELISA	3h	HS vs. IS	0.1 (0-0.47)	0 (0-0)	11	0	6	22	65%	100 %	0.03	ug/l
Rozanski 2017(77)	Prospective study	IS 49, HS (ICH) 25	74	IS 75±10, HS 69±11	not stated	Stroke Emergency Mobile	CT	plasma	ELISA	3h	HS vs. IS	0.057 (0–1.426)	0 (0-0)	9	0	16	49	36%	100 %	0.29	ng/ml
Mattila 2021(105)	Prospective study	IS 178, HS 59	237	IS 70 (61–80), HS 67 (57–76)	IS 111 (54.7), HS 38 (63.3)	ED	NA	plasma	Single-molecule array	3h	HS vs. IS	0.4681 (0.1977-1.1882)	0.1782 (0.0997-0.2951)	57	57	2	121	96.6 %	68%	410	pg/ml
Foerch 2012(149)	Prospective study	IS 163, HS 39	202	IS 75.3±13.4, HS 70.7±17.4	IS 79 (48.5%), HS 21 (55.3%)	Stroke centre	CT; MRI	plasma	Electrochemiluminometric immunoassay	4.5h	HS vs. IS	1.91 (0.41–17.66)	0.08 (0.02–0.14)	33	6	6	157	84.2 %	96.3 %	0.29	ug/l
Foerch 2012(149)	Prospective study	IS 163, HS 39	202	IS 75.3±13.4, HS 70.7±17.4	IS 79 (48.5%), HS 21 (55.3%)	Stroke centre	CT; MRI	plasma	Electrochemiluminometric immunoassay	4.5h	HS vs. IS	1.91 (0.41–17.66)	0.08 (0.02–0.14)	29	2	10	161	73.7 %	98.8 %	0.5	ug/l
Foerch 2012(149)	Prospective study	IS 163, HS 39	202	IS 75.3±13.4, HS 70.7±17.4	IS 79 (48.5%), HS 21 (55.3%)	Stroke centre	CT; MRI	plasma	Electrochemiluminometric immunoassay	4.5h	HS vs. IS	1.91 (0.41–17.66)	0.08 (0.02–0.14)	24	0	15	163	60.5 %	100 %	1	ug/l
Bustamante 2021(168)	Prospective study	IS 154, HS 35	189	IS 81 (70–85), HS 82 (72–85.5)	IS 64 (41.6), HS 20 (57.1)	ED	CT; MRI	plasma	ELISA	4.5h	HS vs. IS	0.045 (0.045–0.059)	error	34	97	1	57	99.4 %	37.1 %	0.05	ng/ml
Ren 2016(82)	Case control study	ICH 45, IS 79	124	ICH 58.91±12.18, IS 61.1±13.33	ICH 36 (80%), IS 49 (62.02%)	ED	CT; MRI	serum	ELISA	4.5h	HS (ICH) vs. IS	0.81 (0.18-3.31)	0.02 (0.004-0.08)	27	3	18	76	61%	96%	0.34	ng/ml
Dvorak 2009(156)	Prospective study	IS 39, HS 14	53	HS 62 (60-70), IS 75 (61- 83)	HS 10 (55.6%), IS 25(55.6%)	NA	CT; MRI	serum	ELISA	6h	HS vs. IS	0.55 (0-1.3)	0 (0-0)	10	4	4	35	71%	95%	0.11	ug/l

Foerch 2006(150)	Prospective study	HS 42, IS 93	135	HS 67±11, IS 70±12	HS 28 (66.7%), IS 57 (61.3%)	stroke unit or NICU	CT; MRI	serum	ELISA	6h	HS vs. IS	0.011 (range 0-3.096)	0 (0-0.014)	33	2	9	91	79%	98%	2.9	ng/l
Katsanos 2017(131)	Prospective study	IS 121, ICH 31	152	IS 77.3±9.1, ICH 74.0±9.6	IS 62 (51.2%), ICH 17 (50.0%)	ED	NA	plasma	BAT GFAP assay	6h	HS (ICH) vs. IS	2.17 (0.55–10.12)	0.11 (0–0.27)	24	4	7	117	91%	97%	0.43	ng/ml
Xiong 2015(47)	Prospective study	IS 65, HS (ICH) 43	108	HS 68.7 ±11.2, IS 70.9 ±9.6	HS 28 (65.1%), IS 44 (67.7%)	NA	CT; MRI	serum	ELISA	6h	HS (ICH) vs. IS	1.6±0.8 (mean± SD)	0.6±0.4(mean± SD)	37	15	6	50	86.0 %	76.9 %	0.7	ng/ml
Unden 2009(57)	Prospective study	IS 83, HS (ICH) 14	97	IS 75 (63,80), HS 64 (60,75)	not stated	NA	CT	NA	ELISA	24h	IS vs. HS (ICH)	0.055 (0.040-0.160)	<0.030 (<0.030-0.070)	11	30	3	53	79%	64%	40	ng/l
Ren 2016(82)	Case control study	ICH 45, IS 79	124	ICH 58.91 ±12.18, IS 61.1 ±13.33	ICH 36 (80%), IS 49 (62.02%)	ED	CT; MRI	serum	ELISA	24h	HS (ICH) vs. IS	0.81 (0.18-3.31)	0.02 (0.004-0.08)	30	7	15	72	67%	91%	0.34	ng/ml

References

1. Zhou X, Qi L. miR-124 Is Downregulated in Serum of Acute Cerebral Infarct Patients and Shows Diagnostic and Prognostic Value. Clin Appl Thromb Hemost 2021;27:10760296211035446.
2. Zhang J, Yang J, Hu J, Zhao W. Clinical value of serum JKAP in acute ischemic stroke patients. Journal of Clinical Laboratory Analysis 2022;36.
3. Yang J, Hao J, Lin Y, Guo Y, Liao K, Yang M, et al. Profile and Functional Prediction of Plasma Exosome-Derived CircRNAs From Acute Ischemic Stroke Patients. Front Genet 2022;13:810974.
4. Turek-Jakubowska A, Debski J, Jakubowski M, Szahidewicz-Krupska E, Gawrys J, Gawrys K, et al. New Candidates for Biomarkers and Drug Targets of Ischemic Stroke-A First Dynamic LC-MS Human Serum Proteomic Study. J Clin Med 2022;11.
5. Tian C, Li Z, Li S, Zhang L, Dai D, Huang Q, et al. The Diagnostic Value of Whole Blood lncRNA NR_120420 for Acute Ischemic Stroke. Oxid Med Cell Longev 2022;2022:1167394.
6. Rahmati M, Ferns GA, Mobarra N. The lower expression of circulating miR-210 and elevated serum levels of HIF-1alpha in ischemic stroke; Possible markers for diagnosis and disease prediction. J Clin Lab Anal 2021;35:e24073.
7. Li G, Han C, Xia X, Yao S. Relationship of uric acid, C-reactive protein, and N-terminal pro-B-type natriuretic peptide with acute cerebral infarction. Revista da Associação Médica Brasileira 2021;67:1639-43.

8. Induruwa I, McKinney H, Kempster C, Thomas P, Batista J, Malcor JD, et al. Platelet surface receptor glycoprotein VI-dimer is overexpressed in stroke: The Glycoprotein VI in Stroke (GYPSIE) study results. PLoS One 2022;17:e0262695.
9. Gawryś K, Turek-Jakubowska A, Gawryś J, Jakubowski M, Dębski J, Szahidewicz-Krupska E, et al. Platelet-Derived Drug Targets and Biomarkers of Ischemic Stroke—The First Dynamic Human LC-MS Proteomic Study. Journal of Clinical Medicine 2022;11.
10. Cho YE, Lee H, Bae HR, Kim H, Yun S, Vorn R, et al. Circulating immune cell landscape in patients who had mild ischaemic stroke. Stroke Vasc Neurol 2022.
11. Intiso D, Zarrelli MM, Lagioia G, Di Rienzo F, Checchia De Ambrosio C, Simone P, et al. Tumor necrosis factor alpha serum levels and inflammatory response in acute ischemic stroke patients. Neurological Sciences 2004;24:390-6.
12. F. Perini MM, M. Alecci, E. Galloni, M. Marchi, V. Toso. Temporal profile of serum anti-inflammatory and pro-inflammatory interleukins in acute ischemic stroke patients. Neurol Sci 2001.
13. Pedersen ED, Waje-Andreassen U, Vedeler CA, Aamodt G, Mollnes TE. Systemic complement activation following human acute ischaemic stroke. Clinical and Experimental Immunology 2004;137:117-22.
14. Nayak AR, Kashyap RS, Kabra D, Purohit HJ, Taori GM, Daginawala HF. Prognostic significance of ischemia-modified albumin in acute ischemic stroke patients: A preliminary study. Ann Neurosci 2011;18:5-7.
15. Mehmet Senes NK, Ozlem Coskun, Oguzhan Zengi, Levent Inan and Dogan Yucel. Oxidative and nitrosative stress in acute ischaemic stroke. Ann Clin Biochem 2007;44:43-7.
16. Feng L, Guo J, Ai F. Circulating long noncoding RNA ANRIL downregulation correlates with increased risk, higher disease severity and elevated pro-inflammatory cytokines in patients with acute ischemic stroke. J Clin Lab Anal 2019;33:e22629.
17. A. Blann PK, J.Krupinski, C. Soluble intercelluar adhesion molecule-1, E-selectin, vascular cell adhesion molecule-1 and von Willebrand factor in stroke. Blood Coagulation and Fibrinolysis 1999;10:277-84.
18. Kou-Gi Shyu HC, Cheng-Chung Lin. Serum levels of intercellular adhesion molecule-1 and E-selectin in patients with acute ischaemic stroke. J Neurol 1997;244:90-3.
19. Liu P, Xiang JW, Jin SX. Serum CXCL12 levels are associated with stroke severity and lesion volumes in stroke patients. Neurol Res 2015;37:853-8.
20. Supanc V, Biloglav Z, Kes VB, Demarin V. Role of cell adhesion molecules in acute ischemic stroke. Ann Saudi Med 2011;31:365-70.
21. Wunderlich MT, Hanhoff T, Goertler M, Spener F, Glatz JF, Wallesch CW, et al. Release of brain-type and heart-type fatty acid-binding proteins in serum after acute ischaemic stroke. J Neurol 2005;252:718-24.
22. Hu Z, Zhu Z, Cao Y, Wang L, Sun X, Dong J, et al. Rapid and Sensitive Differentiating Ischemic and Hemorrhagic Strokes by Dried Blood Spot Based Direct Injection Mass Spectrometry Metabolomics Analysis. J Clin Lab Anal 2016;30:823-30.
23. M Uno KTK, K Nishi, H Itabe, S Nagahiro. Raised plasma oxidised LDL in acute cerebral infarction. J Neurol Neurosurg Psychiatry 2003;74:312–6.
24. Sun D, Tiedt S, Yu B, Jian X, Gottesman RF, Mosley TH, et al. A prospective study of serum metabolites and risk of ischemic stroke. Neurology 2019;92:e1890-e8.

25. Jae Woo Song KSS, Jong Rak Choi, Shin Young Kim, Ji-Hyuk Rhee. Plasma level of IL-6 and its relationship to procoagulant and fibrinolytic markers in acute ischemic stroke. Yonsei Med J Vol 2006;47:201-6.
26. Zitnanova I, Siarnik P, Kollar B, Chomova M, Pazderova P, Andrezalova L, et al. Oxidative Stress Markers and Their Dynamic Changes in Patients after Acute Ischemic Stroke. Oxid Med Cell Longev 2016;2016:9761697.
27. Can S, Akdur O, Yildirim A, Adam G, Cakir DU, Karaman HI. Myelin basic protein and ischemia modified albumin levels in acute ischemic stroke cases. Pak J Med Sci 2015;31:1110-4.
28. Kimberly WT, Wang Y, Pham L, Furie KL, Gerszten RE. Metabolite profiling identifies a branched chain amino acid signature in acute cardioembolic stroke. Stroke 2013;44:1389-95.
29. Abboud H, Labreuche J, Meseguer E, Lavallee PC, Simon O, Olivot JM, et al. Ischemia-modified albumin in acute stroke. Cerebrovasc Dis 2007;23:216-20.
30. Tang Y, Xu H, Du X, Lit L, Walker W, Lu A, et al. Gene expression in blood changes rapidly in neutrophils and monocytes after ischemic stroke in humans: a microarray study. J Cereb Blood Flow Metab 2006;26:1089-102.
31. Rainer TH, Wong KS, Lam W, Lam NY, Graham CA, Lo YM. Comparison of plasma beta-globin DNA and S-100 protein concentrations in acute stroke. Clin Chim Acta 2007;376:190-6.
32. Tiedt S, Duering M, Barro C, Kaya AG, Boeck J, Bode FJ, et al. Serum neurofilament light: A biomarker of neuroaxonal injury after ischemic stroke. Neurology 2018;91:e1338-e47.
33. Xiaoqi Zhu JD, Beiyun Wang, Jie Wang, Min Xu. Circular RNA DLGAP4 is down-regulated and negatively correlates with severity, inflammatory cytokine expression and pro-inflammatory gene miR-143 expression in acute ischemic stroke patients. Int J Clin Exp Pathol 2019;12:941-8.
34. Zhu M, Li N, Luo P, Jing W, Wen X, Liang C, et al. Peripheral Blood Leukocyte Expression of lncRNA MIAT and Its Diagnostic and Prognostic Value in Ischemic Stroke. J Stroke Cerebrovasc Dis 2018;27:326-37.
35. Zhou S, Bao J, Wang Y, Pan S. S100beta as a biomarker for differential diagnosis of intracerebral hemorrhage and ischemic stroke. Neurol Res 2016;38:327-32.
36. Zhou J, Chen L, Chen B, Huang S, Zeng C, Wu H, et al. Increased serum exosomal miR-134 expression in the acute ischemic stroke patients. BMC Neurol 2018;18:198.
37. Zhou X, Xu C, Chao D, Chen Z, Li S, Shi M, et al. Acute Cerebral Ischemia Increases a Set of Brain-Specific miRNAs in Serum Small Extracellular Vesicles. Front Mol Neurosci 2022;15:874903.
38. Zhao X, Yu Y, Xu W, Dong L, Wang Y, Gao B, et al. Apolipoprotein A1-Unique Peptide as a Diagnostic Biomarker for Acute Ischemic Stroke. Int J Mol Sci 2016;17:458.
39. Zhao H, Li G, Ma Q, Tao Z, Wang R, Fan Z, et al. MicroRNA-99a-5p in circulating immune cells as a potential biomarker for the early diagnosis of ischemic stroke. Brain Circ 2017;3:21-8.
40. Zhao B, Zhu Z, Hao J, Wan Z, Guo X. Decreased plasma miR-335 expression in patients with acute ischemic stroke and its association with calmodulin expression. J Int Med Res 2016;44:1331-8.

41. Zhang X, Li Y, Liang Y, Sun P, Wu X, Song J, et al. Distinguishing Intracerebral Hemorrhage from Acute Cerebral Infarction through Metabolomics. Rev Invest Clin 2017;69:319-28.
42. Zhang H, Chen G, Qiu W, Pan Q, Chen Y, Chen Y, et al. Plasma endothelial microvesicles and their carrying miRNA-155 serve as biomarkers for ischemic stroke. J Neurosci Res 2020;98:2290-301.
43. Jarosław Zaremba JL. Interleukin-12 in acute ischemic stroke patients. Folia Neuropathol 2006.
44. Yuan Z, Yu Z, Zhang Y, Yang H. Analysis of the Clinical Diagnostic Value of GMFB in Cerebral Infarction. Curr Pharm Biotechnol 2020;21:955-63.
45. Yigit I, Atescelik M, Yilmaz M, Goktekin MC, Gurger M, Ilhan N. Investigation of UCH-L1 levels in ischemic stroke, intracranial hemorrhage and metabolic disorder induced impaired consciousness. Am J Emerg Med 2017;35:1895-8.
46. Yang ZB, Li TB, Zhang Z, Ren KD, Zheng ZF, Peng J, et al. The Diagnostic Value of Circulating Brain-specific MicroRNAs for Ischemic Stroke. Intern Med 2016;55:1279-86.
47. Xiong L, Yang Y, Zhang M, Xu W. The use of serum glial fibrillary acidic protein test as a promising tool for intracerebral hemorrhage diagnosis in Chinese patients and prediction of the short-term functional outcomes. Neurol Sci 2015;36:2081-7.
48. Wu X, Zhang X, Li D, Zhu Z. Plasma level of miR-99b may serve as potential diagnostic and short-term prognostic markers in patients with acute cerebral infarction. J Clin Lab Anal 2020;34:e23093.
49. Williams JB, Jauch EC, Lindsell CJ, Campos B. Endothelial microparticle levels are similar in acute ischemic stroke and stroke mimics due to activation and not apoptosis/necrosis. Acad Emerg Med 2007;14:685-90.
50. Wang Y, Ma Z, Kan P, Zhang B. The Diagnostic Value of Serum miRNA-221-3p, miRNA-382-5p, and miRNA-4271 in Ischemic Stroke. J Stroke Cerebrovasc Dis 2017;26:1055-60.
51. Wang W, Sun G, Zhang L, Shi L, Zeng Y. Circulating microRNAs as novel potential biomarkers for early diagnosis of acute stroke in humans. J Stroke Cerebrovasc Dis 2014;23:2607-13.
52. Wang W, Li DB, Li RY, Zhou X, Yu DJ, Lan XY, et al. Diagnosis of Hyperacute and Acute Ischaemic Stroke: The Potential Utility of Exosomal MicroRNA-21-5p and MicroRNA-30a-5p. Cerebrovasc Dis 2018;45:204-12.
53. Wang J, Zhao H, Fan Z, Li G, Ma Q, Tao Z, et al. Long Noncoding RNA H19 Promotes Neuroinflammation in Ischemic Stroke by Driving Histone Deacetylase 1-Dependent M1 Microglial Polarization. Stroke 2017;48:2211-21.
54. Walsh KB, Hart K, Roll S, Sperling M, Unruh D, Davidson WS, et al. Apolipoprotein A-I and Paraoxonase-1 Are Potential Blood Biomarkers for Ischemic Stroke Diagnosis. J Stroke Cerebrovasc Dis 2016;25:1360-5.
55. Vukasovic I, Tesija-Kuna A, Topic E, Supanc V, Demarin V, Petrovcic M. Matrix metalloproteinases and their inhibitors in different acute stroke subtypes. Clin Chem Lab Med 2006;44:428-34.
56. von Recum J, Searle J, Slagman A, Vollert JO, Endres M, Mockel M, et al. Copeptin: Limited Usefulness in Early Stroke Differentiation? Stroke Res Treat 2015;2015:768401.
57. Unden J, Strandberg K, Malm J, Campbell E, Rosengren L, Stenflo J, et al. Explorative investigation of biomarkers of brain damage and coagulation system activation in clinical stroke differentiation. J Neurol 2009;256:72-7.

58. Abdulkadir Tunç DK, Mehmet Akdoğan, Saadet Sayan. Serum prolidase enzyme activity as a diagnostic marker for acute ischemic stroke. Biomedical Research 2018;29:1137-42.
59. Tiedt S, Prestel M, Malik R, Schieferdecker N, Duering M, Kautzky V, et al. RNA-Seq Identifies Circulating miR-125a-5p, miR-125b-5p, and miR-143-3p as Potential Biomarkers for Acute Ischemic Stroke. Circ Res 2017;121:970-80.
60. Tiedt S, Brandmaier S, Kollmeier H, Duering M, Artati A, Adamski J, et al. Circulating Metabolites Differentiate Acute Ischemic Stroke from Stroke Mimics. Ann Neurol 2020;88:736-46.
61. Tian D, Zhang S, He X, Liu H. Serum procalcitonin as a diagnostic marker in acute ischemic stroke. Neuroreport 2015;26:33-7.
62. Tian C, Li Z, Yang Z, Huang Q, Liu J, Hong B. Plasma MicroRNA-16 Is a Biomarker for Diagnosis, Stratification, and Prognosis of Hyperacute Cerebral Infarction. PLoS One 2016;11:e0166688.
63. Mohamed A. Shoaeb MAS, Khaled M. Taema, Mohamed A. Hammouda. C-reactive protein A potential biomarker for differentiating ischemic from hemorrhagic stroke. Egypt J Neurol Psychiat Neurosurg 2014;51:311-20.
64. Stejskal D, Sporova L, Svestak M, Karpisek M. Determination of serum visinin like protein-1 and its potential for the diagnosis of brain injury due to the stroke: a pilot study. Biomed Pap Med Fac Univ Palacky Olomouc Czech Repub 2011;155:263-8.
65. Stanca DM, Marginean IC, Soritau O, Dragos C, Marginean M, Muresanu DF, et al. GFAP and antibodies against NMDA receptor subunit NR2 as biomarkers for acute cerebrovascular diseases. J Cell Mol Med 2015;19:2253-61.
66. Stamova B, Xu H, Jickling G, Bushnell C, Tian Y, Ander BP, et al. Gene expression profiling of blood for the prediction of ischemic stroke. Stroke 2010;41:2171-7.
67. Stamova B, Ander BP, Jickling G, Hamade F, Durocher M, Zhan X, et al. The intracerebral hemorrhage blood transcriptome in humans differs from the ischemic stroke and vascular risk factor control blood transcriptomes. J Cereb Blood Flow Metab 2019;39:1818-35.
68. Song H, Zhou H, Qu Z, Hou J, Chen W, Cai W, et al. From Analysis of Ischemic Mouse Brain Proteome to Identification of Human Serum Clusterin as a Potential Biomarker for Severity of Acute Ischemic Stroke. Transl Stroke Res 2019;10:546-56.
69. Simats A, Ramiro L, Garcia-Berrocoso T, Brianso F, Gonzalo R, Martin L, et al. A Mouse Brain-based Multi-omics Integrative Approach Reveals Potential Blood Biomarkers for Ischemic Stroke. Mol Cell Proteomics 2020;19:1921-36.
70. Simats A, Garcia-Berrocoso T, Ramiro L, Giralt D, Gill N, Penalba A, et al. Characterization of the rat cerebrospinal fluid proteome following acute cerebral ischemia using an aptamer-based proteomic technology. Sci Rep 2018;8:7899.
71. Simats A, Garcia-Berrocoso T, Penalba A, Giralt D, Llovera G, Jiang Y, et al. CCL23: a new CC chemokine involved in human brain damage. J Intern Med 2018;283:461-75.
72. Sharma R, Macy S, Richardson K, Lokhnygina Y, Laskowitz DT. A blood-based biomarker panel to detect acute stroke. J Stroke Cerebrovasc Dis 2014;23:910-8.
73. Sharma R, Gowda H, Chavan S, Advani J, Kelkar D, Kumar GS, et al. Proteomic Signature of Endothelial Dysfunction Identified in the Serum of Acute Ischemic Stroke Patients by the iTRAQ-Based LC-MS Approach. J Proteome Res 2015;14:2466-79.
74. MARYAM EMAD SHAKER DZAL. Serum glycogen phosphorylase BB (GPBB) level and Prolidase activity as markers in diagnosis of ischemic stroke. International Journal of Pharmaceutical Research 2020;12.

75. Sepramaniam S, Tan JR, Tan KS, DeSilva DA, Tavintharan S, Woon FP, et al. Circulating microRNAs as biomarkers of acute stroke. Int J Mol Sci 2014;15:1418-32.
76. Sayan S, Kotan D. Levels of brain natriuretic peptide as a marker for the diagnosis and prognosis of acute ischemic stroke. Arch Med Sci Atheroscler Dis 2016;1:e16-e22.
77. Rozanski M, Waldschmidt C, Kunz A, Grittner U, Ebinger M, Wendt M, et al. Glial Fibrillary Acidic Protein for Prehospital Diagnosis of Intracerebral Hemorrhage. Cerebrovasc Dis 2017;43:76-81.
78. Seyed Ali Roudbary FS, Kambiz Forghanparast, and Reza Sohrabnejad. Serum C-reactive protein level as a biomarker for differentiation of ischemic from hemorrhagic stroke. Acta Medica Iranica 2011;49:149-52.
79. Rico Santana N, Zapico Muniz E, Cocho D, Bravo Y, Delgado Mederos R, Marti-Fabregas J. Analysis of peptidome profiling of serum from patients with early onset symptoms of ischemic stroke. J Stroke Cerebrovasc Dis 2014;23:235-40.
80. Richard S, Lapierre V, Girerd N, Bonnerot M, Burkhard PR, Lagerstedt L, et al. Diagnostic performance of peroxiredoxin 1 to determine time-of-onset of acute cerebral infarction. Sci Rep 2016;6:38300.
81. Mark A.Reynolds HJK, Jeffrey R. Dahlen, Joseph M. Anderberg. Early Biomarkers of Stroke. Clinical Chemistry 2003.
82. Ren C, Kobeissy F, Alawieh A, Li N, Li N, Zibara K, et al. Assessment of Serum UCH-L1 and GFAP in Acute Stroke Patients. Sci Rep 2016;6:24588.
83. GS Ranga NB, SB Sharma, R Avasthi. Human carcinoembyronic antigen is a useful biomarker for diagnosis of acute ischemic stroke - A pilot study. Neurology Asia 2016;21:1-6.
84. Rahmati M, Azarpazhooh MR, Ehteram H, Ferns GA, Ghayour-Mobarhan M, Ghannadan H, et al. The elevation of S100B and downregulation of circulating miR-602 in the sera of ischemic stroke (IS) patients: the emergence of novel diagnostic and prognostic markers. Neurol Sci 2020;41:2185-92.
85. Qi Y, Yuan M, Yi Q, Wang Y, Xu L, Xu C, et al. A panel of two miRNAs correlated to systolic blood pressure is a good diagnostic indicator for stroke. Biosci Rep 2021;41.
86. Peycheva M, Deneva T, Zahariev Z. The role of fibrinogen in acute ischaemic stroke. Neurol Neurochir Pol 2021;55:74-80.
87. Perovic E, Mrdjen A, Harapin M, Tesija Kuna A, Simundic AM. Diagnostic and prognostic role of resistin and copeptin in acute ischemic stroke. Top Stroke Rehabil 2017;24:614-8.
88. Penn AM, Saly V, Trivedi A, Lesperance ML, Votova K, Jackson AM, et al. Differential Proteomics for Distinguishing Ischemic Stroke from Controls: a Pilot Study of the SpecTRA Project. Transl Stroke Res 2018;9:590-9.
89. Park SY, Kim MH, Kim OJ, Ahn HJ, Song JY, Jeong JY, et al. Plasma heart-type fatty acid binding protein level in acute ischemic stroke: comparative analysis with plasma S100B level for diagnosis of stroke and prediction of long-term clinical outcome. Clin Neurol Neurosurg 2013;115:405-10.
90. Park KY, Ay I, Avery R, Caceres JA, Siket MS, Pontes-Neto OM, et al. New biomarker for acute ischaemic stroke: plasma glycogen phosphorylase isoenzyme BB. J Neurol Neurosurg Psychiatry 2018;89:404-9.
91. Pan X, Hu Z, Qin L, Han Y, Zhu X, Zhou Y, et al. Applying minimal RNA-seq of peripheral blood platelet mRNA to reveal novel biomarkers in male patients with cerebral stroke. Neuroreport 2020;31:156-61.

92. Palm F, Pussinen PJ, Safer A, Tervahartiala T, Sorsa T, Urbanek C, et al. Serum matrix metalloproteinase-8, tissue inhibitor of metalloproteinase and myeloperoxidase in ischemic stroke. Atherosclerosis 2018;271:9-14.
93. Oraby MI, Rabie RA. Blood biomarkers for stroke: the role of thioredoxin in diagnosis and prognosis of acute ischemic stroke. The Egyptian Journal of Neurology, Psychiatry and Neurosurgery 2019;56.
94. O'Connell GC, Treadway MB, Tennant CS, Lucke-Wold N, Chantler PD, Barr TL. Shifts in Leukocyte Counts Drive the Differential Expression of Transcriptional Stroke Biomarkers in Whole Blood. Transl Stroke Res 2019;10:26-35.
95. O'Connell GC, Stafford P, Walsh KB, Adeoye O, Barr TL. High-Throughput Profiling of Circulating Antibody Signatures for Stroke Diagnosis Using Small Volumes of Whole Blood. Neurotherapeutics 2019;16:868-77.
96. O'Connell GC, Petrone AB, Treadway MB, Tennant CS, Lucke-Wold N, Chantler PD, et al. Machine-learning approach identifies a pattern of gene expression in peripheral blood that can accurately detect ischaemic stroke. NPJ Genom Med 2016;1:16038.
97. O'Connell GC, Petrone AB, Tennant CS, Lucke-Wold N, Kabbani Y, Tarabishy AR, et al. Circulating extracellular DNA levels are acutely elevated in ischaemic stroke and associated with innate immune system activation. Brain Inj 2017;31:1369-75.
98. O'Connell GC, Chantler PD, Barr TL. Stroke-associated pattern of gene expression previously identified by machine-learning is diagnostically robust in an independent patient population. Genom Data 2017;14:47-52.
99. O'Connell GC, Alder ML, Smothers CG, Still CH, Webel AR, Moore SM. Diagnosis of ischemic stroke using circulating levels of brain-specific proteins measured via high-sensitivity digital ELISA. Brain Res 2020;1739:146861.
100. Nielsen HH, Soares CB, Hogedal SS, Madsen JS, Hansen RB, Christensen AA, et al. Acute Neurofilament Light Chain Plasma Levels Correlate With Stroke Severity and Clinical Outcome in Ischemic Stroke Patients. Front Neurol 2020;11:448.
101. Nguyen TTM, van der Bent ML, Wermer MJH, van den Wijngaard IR, van Zwet EW, de Groot B, et al. Circulating tRNA Fragments as a Novel Biomarker Class to Distinguish Acute Stroke Subtypes. Int J Mol Sci 2020;22.
102. Nahan KS, Walsh KB, Adeoye O, Landero-Figueroa JA. The metal and metalloprotein profile of human plasma as biomarkers for stroke diagnosis. J Trace Elem Med Biol 2017;42:81-91.
103. Montaner J, Mendioroz M, Delgado P, Garcia-Berrocoso T, Giralt D, Merino C, et al. Differentiating ischemic from hemorrhagic stroke using plasma biomarkers: the S100B/RAGE pathway. J Proteomics 2012;75:4758-65.
104. Menon B, Ramalingam K, Krishna V. Study of Ischemia Modified Albumin as a Biomarker in Acute Ischaemic Stroke. Ann Neurosci 2018;25:187-90.
105. Mattila OS, Ashton NJ, Blennow K, Zetterberg H, Harve-Rytsala H, Pihlasviita S, et al. Ultra-Early Differential Diagnosis of Acute Cerebral Ischemia and Hemorrhagic Stroke by Measuring the Prehospital Release Rate of GFAP. Clin Chem 2021;67:1361-72.
106. Ryu Matsuo TA, Masahiro Kamouchi, Junya Kuroda, Takahiro Kuwashiro, Jun Hata,Hiroshi Sugimori, Kenji Fukuda, Seiji Gotoh, Noriko Makihara, Masayo Fukuhara, Hideto Awano,, Tetsu Isomura KS, Masahiro Yasaka, Yasushi Okada, Yutaka Kiyohara and Takanari Kitazono. Clinical significance of plasma VEGF value in ischemic stroke - research for biomarkers in ischemic stroke (REBIOS) study. BMC Neurology 2013;13:32.
107. Obata; AMHTJ-e, Ono; STNTK, Ohnishi; MOTMT, Sasayama YDS. Circulating hepatocyte growth factor as a diagnostic marker of thrombus formation in patients with cerebral infarction. Circ J 2002;66:216 –8.

108. Maly M, Hajsl M, Bechynska K, Kucerka O, Sramek M, Suttnar J, et al. Lipidomic Analysis to Assess Oxidative Stress in Acute Coronary Syndrome and Acute Stroke Patients. Metabolites 2021;11.
109. Mahovic D, Zurak N, Lakusic N, Sporis D, Zarkovic N, Stancin N, et al. The dynamics of soluble Fas/APO 1 apoptotic biochemical marker in acute ischemic stroke patients. Adv Med Sci 2013;58:298-303.
110. Ma Q, Li G, Tao Z, Wang J, Wang R, Liu P, et al. Blood microRNA-93 as an indicator for diagnosis and prediction of functional recovery of acute stroke patients. J Clin Neurosci 2019;62:121-7.
111. Luger S, Witsch J, Dietz A, Hamann GF, Minnerup J, Schneider H, et al. Glial Fibrillary Acidic Protein Serum Levels Distinguish between Intracerebral Hemorrhage and Cerebral Ischemia in the Early Phase of Stroke. Clin Chem 2017;63:377-85.
112. Lu D, Ho ES, Mai H, Zang J, Liu Y, Li Y, et al. Identification of Blood Circular RNAs as Potential Biomarkers for Acute Ischemic Stroke. Front Neurosci 2020;14:81.
113. Guangwen Long FW, Huaping Li, Zhongwei Yin, Chaugai Sandip, Yan Lou, Yan Wang, Chen Chen, Dao Wen Wang. Circulating miR-30a, miR-126 and let-7b as biomarker for ischemic stroke in humans. BMC Neurology 2013;13:178.
114. Llombart V, Garcia-Berrocoso T, Bustamante A, Giralt D, Rodriguez-Luna D, Muchada M, et al. Plasmatic retinol-binding protein 4 and glial fibrillary acidic protein as biomarkers to differentiate ischemic stroke and intracerebral hemorrhage. J Neurochem 2016;136:416-24.
115. Liu Y, Zhang J, Han R, Liu H, Sun D, Liu X. Downregulation of serum brain specific microRNA is associated with inflammation and infarct volume in acute ischemic stroke. J Clin Neurosci 2015;22:291-5.
116. Liu P, Li R, Antonov AA, Wang L, Li W, Hua Y, et al. Discovery of Metabolite Biomarkers for Acute Ischemic Stroke Progression. J Proteome Res 2017;16:773-9.
117. Liu J, Sugimoto K, Cao Y, Mori M, Guo L, Tan G. Serum Sphingosine 1-Phosphate (S1P): A Novel Diagnostic Biomarker in Early Acute Ischemic Stroke. Front Neurol 2020;11:985.
118. Liswati E, Wijaya A, Ranakusuma TAS. Biochemical Markers for Differential Diagnosis of Stroke: A Biochemical Markers Study of S100B Protein, Neuron Spesific Enolase (NSE), Myelin Basic Protein (MBP), and Heart-Type Fatty Acid Binding Protein (H-FABP). The Indonesian Biomedical Journal 2009;1.
119. Li X, Xu L, Xu Z. The diagnostic and prognostic performance of Lp-PLA2 in acute ischemic stroke. Med Clin (Barc) 2021;156:437-43.
120. Li P, Teng F, Gao F, Zhang M, Wu J, Zhang C. Identification of circulating microRNAs as potential biomarkers for detecting acute ischemic stroke. Cell Mol Neurobiol 2015;35:433-47.
121. Li G, Ma Q, Wang R, Fan Z, Tao Z, Liu P, et al. Diagnostic and Immunosuppressive Potential of Elevated Mir-424 Levels in Circulating Immune Cells of Ischemic Stroke Patients. Aging Dis 2018;9:172-81.
122. Leung LY, Chan CP, Leung YK, Jiang HL, Abrigo JM, Wang de F, et al. Comparison of miR-124-3p and miR-16 for early diagnosis of hemorrhagic and ischemic stroke. Clin Chim Acta 2014;433:139-44.
123. Laterza OF, Modur VR, Crimmins DL, Olander JV, Landt Y, Lee JM, et al. Identification of novel brain biomarkers. Clin Chem 2006;52:1713-21.
124. Laskowitz DT, Kasner SE, Saver J, Remmel KS, Jauch EC, Group BS. Clinical usefulness of a biomarker-based diagnostic test for acute stroke: the Biomarker Rapid Assessment in Ischemic Injury (BRAIN) study. Stroke 2009;40:77-85.

125. Danuta Kokocińska PW, Robert Partyka, Jerzy Jarząb, Przemysław Jałowiecki & Jerzy Sikora The diagnostic utility of S-100B protein and TPA in patients with ischemic stroke. Neuroendocrinology Letters 2007;28:693–8.
126. Danuta Kokocińska PG, Przemysław Jałowiecki, Robert Partyka,, Piotr Wieczorek ŁM, Izabela Chanek, Jerzy Jarząb, Lech Cierpka. The usefulness of assessing the serum levels of S- 100 protein in patients with ischemic stroke. Acta Angiol 2005;11:105-13.
127. Kodali P, Landero-Figueroa JA, Caruso J, Adeoye O. A preliminary method development study to identify potential stroke biomarkers in plasma using multiple chromatographies with nanoLC-ESIMS detection. J Neural Transm (Vienna) 2013;120:1441-5.
128. Kodali P, Chitta KR, Landero Figueroa JA, Caruso JA, Adeoye O. Detection of metals and metalloproteins in the plasma of stroke patients by mass spectrometry methods. Metallomics 2012;4:1077-87.
129. Jan Kochanowski MG, Agnieszka Baranowska-Bik,, Ewa Wolinska-Witort MK, Boguslawa Baranowska,,Wojciech Bik. Resistin levels in women with ischemic stroke. Neuroendocrinol Lett 2012;33:603-7.
130. Kavalci C GH, Durukan P, Cevik Y. Value of biomarker-based diagnostic test in differential diagnosis of hemorrhagic-ischemic stroke. Bratisl Lek Listy 2011;112:398-401.
131. Katsanos AH, Makris K, Stefani D, Koniari K, Gialouri E, Lelekis M, et al. Plasma Glial Fibrillary Acidic Protein in the Differential Diagnosis of Intracerebral Hemorrhage. Stroke 2017;48:2586-8.
132. Kashyap RS, Nayak AR, Deshpande PS, Kabra D, Purohit HJ, Taori GM, et al. Inter-alpha-trypsin inhibitor heavy chain 4 is a novel marker of acute ischemic stroke. Clin Chim Acta 2009;402:160-3.
133. Kara H, Akinci M, Degirmenci S, Bayir A, Ak A, Nayman A, et al. High-sensitivity C-reactive protein, lipoprotein-related phospholipase A2, and acute ischemic stroke. Neuropsychiatr Dis Treat 2014;10:1451-7.
134. Kalra LP, Khatter H, Ramanathan S, Sapehia S, Devi K, Kaliyaperumal A, et al. Serum GFAP for stroke diagnosis in regions with limited access to brain imaging (BE FAST India). Eur Stroke J 2021;6:176-84.
135. Kalani MYS, Alsop E, Meechoovet B, Beecroft T, Agrawal K, Whitsett TG, et al. Extracellular microRNAs in blood differentiate between ischaemic and haemorrhagic stroke subtypes. J Extracell Vesicles 2020;9:1713540.
136. Jin F, Xing J. Circulating pro-angiogenic and anti-angiogenic microRNA expressions in patients with acute ischemic stroke and their association with disease severity. Neurol Sci 2017;38:2015-23.
137. Jiang Z, Sun J, Liang Q, Cai Y, Li S, Huang Y, et al. A metabonomic approach applied to predict patients with cerebral infarction. Talanta 2011;84:298-304.
138. Jia L, Hao F, Wang W, Qu Y. Circulating miR-145 is associated with plasma high-sensitivity C-reactive protein in acute ischemic stroke patients. Cell Biochem Funct 2015;33:314-9.
139. Ji Q, Ji Y, Peng J, Zhou X, Chen X, Zhao H, et al. Increased Brain-Specific MiR-9 and MiR-124 in the Serum Exosomes of Acute Ischemic Stroke Patients. PLoS One 2016;11:e0163645.
140. Inoue T, Ishida T, Inoue T, Saito A, Ezura M, Uenohara H, et al. Lectin-Like Oxidized Low-Density Lipoprotein Receptor-1 Levels as a Biomarker of Acute Intracerebral Hemorrhage. J Stroke Cerebrovasc Dis 2019;28:490-4.
141. Iltumur K, Yavavli A, Apak I, Ariturk Z, Toprak N. Elevated plasma N-terminal pro-brain natriuretic peptide levels in acute ischemic stroke. Am Heart J 2006;151:1115-22.

142. Herisson F, Delaroche O, Auffray-Calvier E, Duport BD, Guillon B. Ischemia-modified albumin and heart fatty acid-binding protein: could early ischemic cardiac biomarkers be used in acute stroke management? J Stroke Cerebrovasc Dis 2010;19:279-82.
143. Han K, Jia N, Yang L, Min LQ. Correlation between ischemia-modified albumin and lipid levels in patients with acute cerebrovascular disease. Mol Med Rep 2012;6:621-4.
144. Gunduz A, Turedi S, Mentese A, Altunayoglu V, Turan I, Karahan SC, et al. Ischemia-modified albumin levels in cerebrovascular accidents. Am J Emerg Med 2008;26:874-8.
145. Gunaydin M, Turkmen S, Sahin A, Sumer A, Mentese A, Turedi S, et al. The diagnostic value of SCUBE1 levels in acute ischemic stroke. Turkish Journal of Biochemistry 2014;39:107-12.
146. Glickman SW, Phillips S, Anstrom KJ, Laskowitz DT, Cairns CB. Discriminative capacity of biomarkers for acute stroke in the emergency department. J Emerg Med 2011;41:333-9.
147. Sotirios Giannopoulos MK, Apostolos I. Hatzitolios,, Christos G. Savopoulos AZ, Michail Karamouzis. Measurements of endothelin-1, C-reactive protein and fibrinogen plasma levels in patients with acute ischemic stroke. Neurol Res 2008;30:727–30.
148. Garlichs CD, Kozina S, Fateh-Moghadam S, Handschu R, Tomandl B, Stumpf C, et al. Upregulation of CD40-CD40 ligand (CD154) in patients with acute cerebral ischemia. Stroke 2003;34:1412-8.
149. Foerch C, Niessner M, Back T, Bauerle M, De Marchis GM, Ferbert A, et al. Diagnostic accuracy of plasma glial fibrillary acidic protein for differentiating intracerebral hemorrhage and cerebral ischemia in patients with symptoms of acute stroke. Clin Chem 2012;58:237-45.
150. Foerch C, Curdt I, Yan B, Dvorak F, Hermans M, Berkefeld J, et al. Serum glial fibrillary acidic protein as a biomarker for intracerebral haemorrhage in patients with acute stroke. J Neurol Neurosurg Psychiatry 2006;77:181-4.
151. U. Fiszer GK-K, W. Palasik JK, A. Gorski,, Wonkowska A. Increased expression of adhesion molecule CD18 (LFA-1beta) on the leukocytes of peripheral blood in patients with acute ischemic stroke. Acta Neurol Scand 1998;97:221-4.
152. Klaus Fassbender RS, Andreas Schreiner, Mark Fatar, Frank Muhlhauser,, Michael Daffertshofer MH. Leakage of brain-originated proteins in peripheral blood temporal profile and diagnostic value in early ischemic stroke. Journal of Neurological Sciences 1997;148:105-9.
153. Fang C, Lou B, Zhou J, Zhong R, Wang R, Zang X, et al. Blood biomarkers in ischemic stroke: Role of biomarkers in differentiation of clinical phenotype. European Journal of Inflammation 2018;16.
154. Ewida HA, Zayed RK, Darwish HA, Shaheen AA. Circulating lncRNAs HIF1A-AS2 and LINLK-A: Role and Relation to Hypoxia-Inducible Factor-1alpha in Cerebral Stroke Patients. Mol Neurobiol 2021;58:4564-74.
155. Ekingen E, Yilmaz M, Yildiz M, Atescelik M, Goktekin MC, Gurger M, et al. Utilization of glial fibrillary acidic protein and galectin-3 in the diagnosis of cerebral infarction patients with normal cranial tomography. Niger J Clin Pract 2017;20:433-7.
156. Dvorak F, Haberer I, Sitzer M, Foerch C. Characterisation of the diagnostic window of serum glial fibrillary acidic protein for the differentiation of intracerebral haemorrhage and ischaemic stroke. Cerebrovasc Dis 2009;27:37-41.
157. Duan XX, Zhang GP, Wang XB, Yu H, Wu JL, Liu KZ, et al. The diagnostic and prognostic value of serum CXCL12 levels in patients with ischemic stroke. Neurol Sci 2015;36:2227-34.
158. De Marchis GM, Katan M, Barro C, Fladt J, Traenka C, Seiffge DJ, et al. Serum neurofilament light chain in patients with acute cerebrovascular events. Eur J Neurol 2018;25:562-8.

159. Dassan P, Keir G, Jager HR, Brown MM. Value of measuring serum vascular endothelial growth factor levels in diagnosing acute ischemic stroke. Int J Stroke 2012;7:454-9.
160. Dambinova. Blood test detecting autoantibodies to N-methyl-D-aspartate neuroreceptors for evaluation of patients with transient ischemic attack and stroke. 2003.
161. Dambinova SA, Bettermann K, Glynn T, Tews M, Olson D, Weissman JD, et al. Diagnostic potential of the NMDA receptor peptide assay for acute ischemic stroke. PLoS One 2012;7:e42362.
162. Cheng X, Kan P, Ma Z, Wang Y, Song W, Huang C, et al. Exploring the potential value of miR-148b-3p, miR-151b and miR-27b-3p as biomarkers in acute ischemic stroke. Biosci Rep 2018;38.
163. Chen Z, Wang K, Huang J, Zheng G, Lv Y, Luo N, et al. Upregulated Serum MiR-146b Serves as a Biomarker for Acute Ischemic Stroke. Cell Physiol Biochem 2018;45:397-405.
164. Cavrak ME, Hass R, Stephens RJ, Adcock A, Petrone AB. Leukocyte Biomarkers for the Differential Diagnosis of Mild Acute Ischemic Stroke, Transient Ischemic Attack, and Stroke Mimic. Cureus 2021;13:e13383.
165. Climaco P. Cano VPBd, Herna´n E. Atencio, Mayerlim T. Medina, , Amell Anilsa AS, Omaira M. Molina, Helen Restrepo, Maria E. Vargas. Increased serum malondialdehyde and decreased nitric oxide within 24 hours of thrombotic stroke onset. American Journal of Therapeutics 2003;10:473–6.
166. Altunayoglu Cakmak V, Gunduz A, Karaca Y, Alioglu Z, Mentese A, Topbas M. Diagnostic Significance of Ischemia-Modified Albumin, S100b, and Neuron-Specific Enolase in Acute Ischemic Stroke. Journal of Academic Emergency Medicine 2014.
167. Buttner T, Weyers S, Postert T, Sprengelmeyer R, Kuhn W. S-100 protein: Serum marker of focal brain damage after ischemic territorial MCA infarction. Stroke 1997;28:1961-5.
168. Bustamante A, Penalba A, Orset C, Azurmendi L, Llombart V, Simats A, et al. Blood Biomarkers to Differentiate Ischemic and Hemorrhagic Strokes. Neurology 2021;96:e1928-e39.
169. Bustamante A, Lopez-Cancio E, Pich S, Penalba A, Giralt D, Garcia-Berrocoso T, et al. Blood Biomarkers for the Early Diagnosis of Stroke: The Stroke-Chip Study. Stroke 2017;48:2419-25.
170. Bolayir A, Gokce SF, Cigdem B, Bolayir HA, Gulunay A, Celik VK, et al. Can SCUBE1 be used to predict the early diagnosis, lesion volume and prognosis of acute ischemic stroke? Turkish Journal of Biochemistry 2019;44:16-24.
171. Bibl M, Welge V, Schmidt H, Esselmann H, Mollenhauer B, Lewczuk P, et al. Plasma amyloid-beta peptides in acute cerebral ischemia: a pilot study. J Clin Lab Anal 2012;26:238-45.
172. T.L. Barr, Y. Conley, J. Ding, A. Dillman, S. Warach, A. Singleton, et al. Genomic biomarkers and cellular pathways of ischemic stroke by RNA gene expression profiling. Neurology 2010;75:1009-14.
173. Azarpazhooh MR, Mobarra N, Parizadeh SM, Tavallaie S, Bagheri M, Rahsepar AA, et al. Serum high-sensitivity C-reactive protein and heat shock protein 27 antibody titers in patients with stroke and 6-month prognosis. Angiology 2010;61:607-12.
174. Atik I, Kozaci N, Beydilli I, Avci M, Ellidag H, Kesapli M. Investigation of oxidant and antioxidant levels in patients with acute stroke in the emergency service. Am J Emerg Med 2016;34:2379-83.

175. Alvarez-Perez FJ, Castelo-Branco M, Alvarez-Sabin J. Usefulness of measurement of fibrinogen, D-dimer, D-dimer/fibrinogen ratio, C reactive protein and erythrocyte sedimentation rate to assess the pathophysiology and mechanism of ischaemic stroke. J Neurol Neurosurg Psychiatry 2011;82:986-92.
176. Allard L, Lescuyer P, Burgess J, Leung KY, Ward M, Walter N, et al. ApoC-I and ApoC-III as potential plasmatic markers to distinguish between ischemic and hemorrhagic stroke. Proteomics 2004;4:2242-51.
177. Allard L, Burkhard PR, Lescuyer P, Burgess JA, Walter N, Hochstrasser DF, et al. PARK7 and nucleoside diphosphate kinase A as plasma markers for the early diagnosis of stroke. Clin Chem 2005;51:2043-51.
178. Algin A, Erdogan MO, Aydin I, Poyraz MK, Sirik M. Clinical usefulness of brain-derived neurotrophic factor and visinin-like protein-1 in early diagnostic tests for acute stroke. Am J Emerg Med 2019;37:2051-4.
179. Gheyath AlGawwam ZaI, Maryam Emad Shake. Evaluation of glycogen phosphorylase bb (GPBB) level in serum as marker in diagnosis of acute ischemic stroke. Indian Journal of Forensic Medicine & Toxicology 2021;1603-1608.
180. Ahn JH, Choi SC, Lee WG, Jung YS. The usefulness of albumin-adjusted ischemia-modified albumin index as early detecting marker for ischemic stroke. Neurol Sci 2011;32:133-8.
181. Adly Sadik N, Ahmed Rashed L, Ahmed Abd-El Mawla M. Circulating miR-155 and JAK2/STAT3 Axis in Acute Ischemic Stroke Patients and Its Relation to Post-Ischemic Inflammation and Associated Ischemic Stroke Risk Factors. Int J Gen Med 2021;14:1469-84.
182. Abe A, Tanaka M, Yasuoka A, Saito Y, Okada S, Mishina M, et al. Changes in Whole-Blood microRNA Profiles during the Onset and Treatment Process of Cerebral Infarction: A Human Study. Int J Mol Sci 2020;21.
